# Perspectives on low-value care and barriers to de-implementation among primary care physicians: a multinational survey

**DOI:** 10.1186/s12875-024-02382-9

**Published:** 2024-05-09

**Authors:** Aleksi Raudasoja, Kari A. O. Tikkinen, Benedetta Bellini, Eliana Ben-Sheleg, Moriah E Ellen, Paolo Francesconi, Muaad Hussien, Yuki Kaji, Eleni Karlafti, Shunzo Koizumi, Emir Ouahrani, Muna Paier-Abuzahra, Christos Savopoulos, Ulrike Spary-Kainz, Jorma Komulainen, Raija Sipilä

**Affiliations:** 1 Faculty of Medicine, University of Helsinki, Helsinki, Finland; 2 Department of Urology, University of Helsinki and Helsinki University Hospital, Helsinki, Finland; 3 Department of Surgery, South Karelian Central Hospital, Lappeenranta, Finland; 4 Regional Health Agency, Florence, Italy; 5 Department of Epidemiology, Biostatistics and Community Health Sciences, University of the Negev, Be’er Sheva, Israel; 6 Israel Implementation Science and Policy Engagement Centre (IS-PEC), Ben-Gurion University of the Negev, Be’er Sheva, Israel; 7 Department of Health Policy and Management, and Israel Implementation Science and Policy Engagement Centre (IS-PEC), Ben-Gurion University of the Negev, Be’er Sheva, Israel; 8 Institute of Health Policy Management and Evaluation, Dalla Lana School of Public Health, University of Toronto, Toronto, Canada; 9 Department of Medicine, Mälarsjukhuset Hospital, Eskilstuna, Sweden; 10 Department of General Medicine, Division of Behavioral Sciences, International University of Health and Welfare Narita Hospital, Narita, Japan; 11 Emergency Department, and 1st Propedeutic Department of Internal Medicine, AHEPA University Hospital, Aristotle University of Thessaloniki, Thessaloniki, Greece; 12 Shichijo Clinic, Saga Medical School, Kyoto, Saga, Japan; 13 Department of geriatrics, Karolinska University Hospital, Stockholm, Sweden; 14 Institute of General Practice and Evidence-Based Health Services Research, Medical University of Graz, Graz, Austria; 15 1st Propedeutic Department of Internal Medicine, AHEPA University Hospital, Aristotle University of Thessaloniki, Thessaloniki, Greece; 16 Finnish Medical Society Duodecim, Helsinki, Finland; 17 Department of Health Research Methods, Evidence and Impact, McMaster University, Hamilton, Canada

**Keywords:** De-implementation, Low-value care, Barriers and facilitators, Overdiagnosis, Overtreatment, Complex interventions

## Abstract

**Background:**

Healthcare costs are rising worldwide. At the same time, a considerable proportion of care does not benefit or may even be harmful to patients. We aimed to explore attitudes towards low-value care and identify the most important barriers to the de-implementation of low-value care use in primary care in high-income countries.

**Methods:**

Between May and June 2022, we email surveyed primary care physicians in six high-income countries (Austria, Finland, Greece, Italy, Japan, and Sweden). Physician respondents were eligible if they had worked in primary care during the previous 24 months. The survey included four sections with categorized questions on (1) background information, (2) familiarity with Choosing Wisely recommendations, (3) attitudes towards overdiagnosis and overtreatment, and (4) barriers to de-implementation, as well as a section with open-ended questions on interventions and possible facilitators for de-implementation. We used descriptive statistics to present the results.

**Results:**

Of the 16,935 primary care physicians, 1,731 answered (response rate 10.2%), 1,505 had worked in primary care practice in the last 24 months and were included in the analysis. Of the respondents, 53% had read Choosing Wisely recommendations. Of the respondents, 52% perceived overdiagnosis and 50% overtreatment as at least a problem to some extent in their own practice. Corresponding figures were 85% and 81% when they were asked regarding their country’s healthcare. Respondents considered patient expectations (85% answered either moderate or major importance), patient’s requests for treatments and tests (83%), fear of medical error (81%), workload/lack of time (81%), and fear of underdiagnosis or undertreatment (79%) as the most important barriers for de-implementation. Attitudes and perceptions of barriers differed significantly between countries.

**Conclusions:**

More than 80% of primary care physicians consider overtreatment and overdiagnosis as a problem in their country’s healthcare but fewer (around 50%) in their own practice. Lack of time, fear of error, and patient pressures are common barriers to de-implementation in high-income countries and should be acknowledged when planning future healthcare. Due to the wide variety of barriers to de-implementation and differences in their importance in different contexts, understanding local barriers is crucial when planning de-implementation strategies.

**Supplementary Information:**

The online version contains supplementary material available at 10.1186/s12875-024-02382-9.

## Introduction

The American Board of Internal Medicine (ABIM) launched the Choosing Wisely (CW) campaign in 2012. The aim was to increase discussions between clinicians and patients to achieve better care and especially reduce use of unnecessary care [1]. Low-value care, previously defined as care that (1) provides no benefit, (2) wastes limited resources and (3) potentially causes harm to the patient [2], has gained more attention in the last decade. ABIM has since terminated the campaign but Choosing Wisely campaigns have been implemented in several other countries.

In a survey to members of the American Medical Association in 2014, physicians estimated 21% of overall medical care to be unnecessary [3]. Current estimates about US healthcare waste range between 21 and 47%, and overtreatment has been estimated to be approximately 2.5% of total healthcare costs in the US [4, 5]. In a Dutch survey among primary care physicians, as many as 67% of the respondents thought that “low-value care practices are regularly provided in the health care” [6]. Although the exact prevalence of low-value care may be impossible to estimate [7], increasing evidence suggests that large number of specific low-value practices are prevalent in modern healthcare [8–10].

De-implementation refers to strategies to reduce the use of low-value care. Understanding the barriers to change is an important part of designing implementation and de-implementation strategies [11, 12]. Earlier evidence suggests that both barriers related to individual providers, such as attitudes, knowledge and skills, and patient-related barriers, such as patients’ preferences, expectations and requests are important in de-implementation [13].

Although earlier studies have suggested potential barriers to de-implementation, it remains uncertain which barriers indeed are the most important. Low-value care use is common and widely recognized in primary care [14, 15]. We therefore surveyed the most important barriers to de-implementation in primary care. In our multinational survey among primary care physicians, we also explored potential differences in attitudes towards low-value care.

## Methods

We developed a survey in collaboration with a multinational team (Austria, Finland, Greece, Italy, Japan, and Sweden), including primary care physicians and research methodologists. We included only high-income countries from which we found local investigators that could participate in the data collection and who had a comprehensive list of emails for primary care physicians available. We especially sought collaboration among international Choosing Wisely network. The survey included five sections: (1) background information, (2) familiarity with Choosing Wisely recommendations, (3) attitudes towards overdiagnosis and overtreatment, (4) barriers to de-implementation, (5) interventions, and possible facilitators for de-implementation. We explained all concepts used in the survey, by using definitions in the previous literature [16, 17]. Overdiagnosis was explained as “1) the diagnosis of a medical condition that would never cause any symptoms or problems, or 2) medicalizing ordinary life experiences through expanded definitions of diseases. Overdiagnosis can be caused by overdetection or overdefinition of disease”. Overtreatment was explained as “treatment for which there is no or little benefit to the patient, considering both the potential harm from and benefit of the treatment”. We piloted the survey in Finland (five primary care physicians and one layperson) and asked comments from two content experts. The survey was translated by professional translators in every country and reviewed by local researchers (translations of the survey are available in the related file).

As background information, respondents provided their age, work experience, specialization status, and gender. In sections two to four, we used a four-point Likert scale [18] (Table 1).

**Table 1:** Survey questions and response options

Question (questionnaire section)	Response options
Are you familiar with the Choosing Wisely recommendations? (Sect. 2)	I have never heard of them / I have heard of them / I have read a few / I have read many
Do you follow Choosing Wisely recommendations which are relevant in your own clinical practice? (Sect. 2)	Never / Rarely / Often / Always
In my practice / In my country’s healthcare system / In other high income countries, overdiagnosis is ____? (Sect. 3)	Not problem at all / A minor problem / A problem to some extent / A major problem
In my practice / In my country’s healthcare system / In other high income countries, overtreatment is ____? (Sect. 3)	Not problem at all / A minor problem / A problem to some extent / A major problem
How important each listed individual barrier is in your own clinical practice? (Sect. 4)	No importance / Small importance / Moderate importance / Major importance

We used previous literature to develop a list of potential barriers [3, 6, 13, 14, 19–21]. In section four, participants were asked to evaluate how important each listed barrier is in their own clinical practice. We used three categories for the barriers: individual, organizational, and patient-related barriers.

The last section of the survey, which explored the most important low-value care practices and potential interventions to facilitate de-implementation, consisted of open-ended questions. These results will be presented elsewhere.

### Sample

In each participating country, we aimed to send the survey to a random sample of at least 2000 primary care physicians who were active in clinical practice. Japan, Sweden, Austria, and Italy included all primary care physicians which led to larger samples than 2000. In collaboration with the primary investigator (AR), the local researchers aimed to create as representative samples as possible. We included respondents if they had worked in primary care in the previous 24 months. The e-mails were sent by local medical societies or medical societies provided the e-mail lists to the local investigators (except in Sweden, where emails were sent by a private company [IQVIA Solutions Sweden AB]). Invitations to participate in the survey were written in the local language. Sample formulation for each country is reported in the Supplementary file (S Tables 1, 2, 3, 4, 5 and 6).

### Procedure

We sent an email one week before the first round to inform potential respondents about the upcoming survey. We conducted the e-mail survey between May and June 2022 (except in Japan, November-December 2022). After the invitation letter and personal link to the questionnaire, all participants received two email reminders at two-week intervals. To collect the responses, we used SurveyMonkey in Finland, Greece, Japan, and Sweden, Limesurvey in Austria, and a locally created platform in Italy. Responses were collected anonymously.

### Analysis

We used descriptive statistics to present the survey responses. For each question in sections two to four, we calculated the proportion of respondents answering different options on the four-point Likert scale. In addition, we calculated the proportions of respondents who rated overtreatment and overdiagnosis as a bigger problem for themselves, a bigger problem for their country’s healthcare, or as big of a problem for themselves as for their country’s healthcare. For barriers to de-implementation, we recategorized the four-point scale into a two-point scale: no/minor importance or moderate/major importance, and present the proportions of responding moderate/major importance.

In the secondary analyses, we used Survey package in R statistics [22]. We used the country as a stratum and formed a general linear model with work experience, gender, Choosing Wisely familiarity, and attitudes towards overdiagnosis and overtreatment as covariates. We used one-way ANOVA to test differences between groups and the effects of covariates.

Substantial differences in the mean importance of all barriers suggested that there could be response bias. Therefore, after the main analysis, we compared the country mean of each barrier to the country-specific mean of all barriers.

## Results

Of the 16,935 primary care physicians, 1,731 responded (response rate 10,2%). Response rates ranged from 5,5% (Japan) to 22% (Finland). Of the 1,731 respondents, 1505 had worked in primary care practice in the last 24 months and responded to at least one analyzed question and were therefore included in the analysis. Over 60% of the respondents had at least ten years of work experience and 53% were male (Table 2). About half of the respondents (56%) were specialists in family medicine; 19% were specialists in other areas. Differences between countries represented the local primary care system, e.g. hospital medicine specialists in Japan and internal medicine specialists in Greece working in primary care, who mostly represented the “other” category in these countries (S Tables 7, 8, 9, 10, 11, 12, 13, 14 and 15). The Remaining 25% were either specializing (44% of these were specializing/in training in family medicine) or did not have specialization (worked in primary care without formal training leading to specialization).

**Table 2:** Respondent characteristics

	Total *N* = 1,505		Austria *N* = 238	Finland *N* = 370	Greece *N* = 95	Italy *N* = 246	Japan *N* = 280	Sweden *N* = 276
Work experience (years)	%	n	%	%	%	%	%	%
Under 5 5–10 11–20 21–30 Over 30	17 21 26 20 16	258 316 385 298 245	17 22 28 14 19	30 19 23 14 15	4 13 40 35 8	24 15 9 28 25	10 30 38 14 8	7 23 25 26 20
Gender*								
Male Female	53 46	803 698	52 47	39 61	54 46	61 39	72 28	47 53
Specialization								
Specialist in FM Specialist - other Specializing/no specialization	56 19 25	840 289 376	99 1 0	34 9 56	17 80 3	50 28 22	47 34 19	75 3 21

FM = family medicine

*In addition, 1 “other” response, and 3 respondents with a missing answer

Country-specific characteristics had big differences, although they represented the primary care physician population distributions in each country (S Tables 7, 8, 9, 10, 11, 12, 13, 14 and 15). For example, in Finland, specializing to any area of medicine requires nine months of work in primary care which leads to younger physician population than in other countries.

Of the 1,505 respondents, 66 dropped out after Choosing Wisely familiarity questions (1,439 responses remained), and 153 after the questions regarding attitudes. Finally, after excluding 66 respondents who answered having no problems both with overdiagnosis and overtreatment in their own practice, 1,220 responded to barrier questions.

Of 1,505 respondents, 26.5% had never heard of Choosing Wisely recommendations, 20.2% had heard of them, 32.3% had read a few, and 21.0% had read many of them. Of the 800 (53.1%) respondents that had read at least a few Choosing Wisely recommendations, 72.1% reported often following the recommendations relevant to their own practice, and 14.1% always. The familiarity with Choosing Wisely recommendations (proportion of respondents who had read at least a few CW recommendations) varied between countries, from 18.1% in Austria and 23.2% in Greece to 70.5% in Finland and 79.0% in Sweden.

**Fig. 1:**
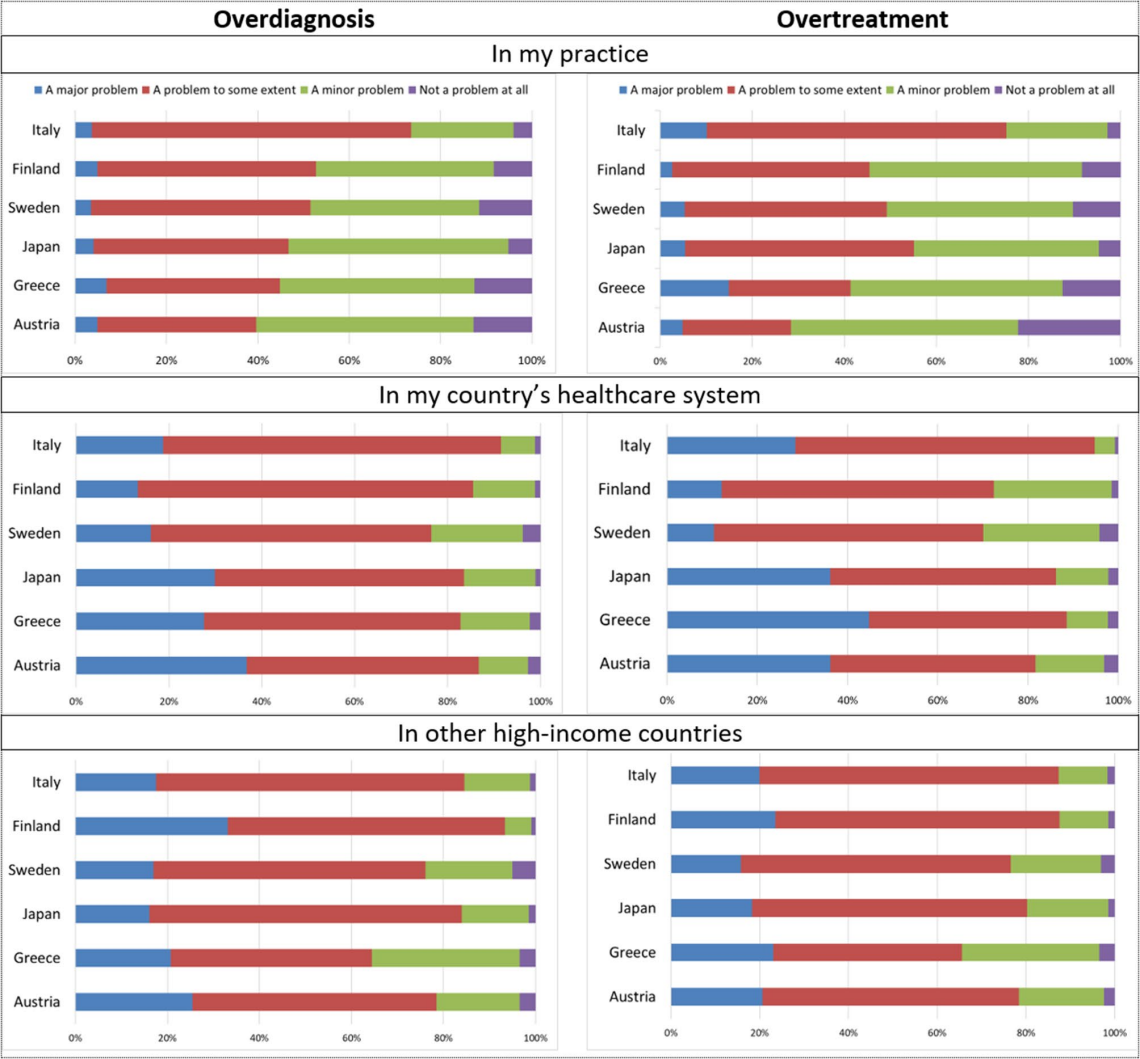
Attitudes toward overdiagnosis and overtreatment

**Fig. 2:**
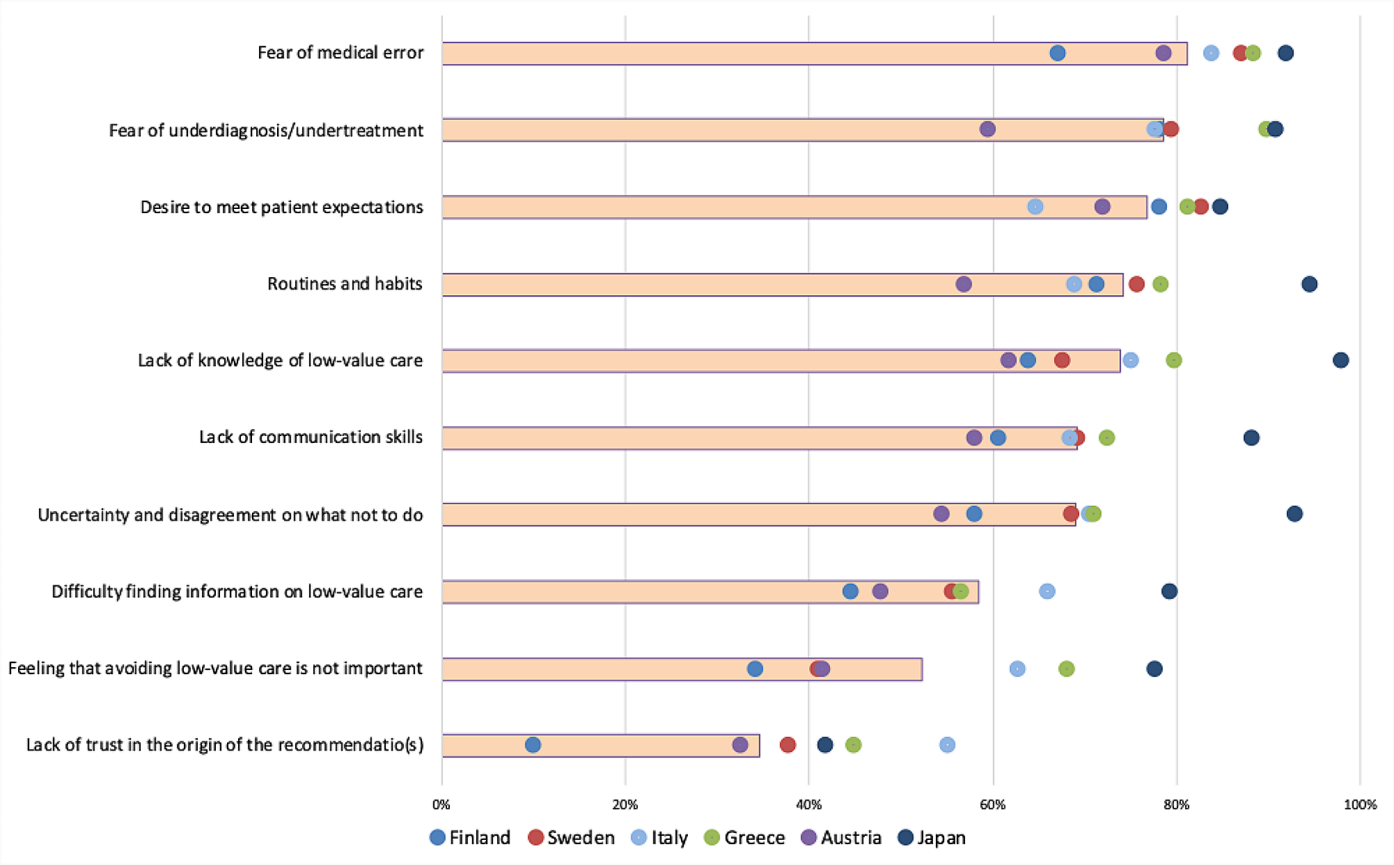
Individual barriers: proportion (%) of moderate or major importance responses. *The bars represent the proportion of moderate/major responses of all responses and the dots country-specific proportions*

**Fig. 3:**
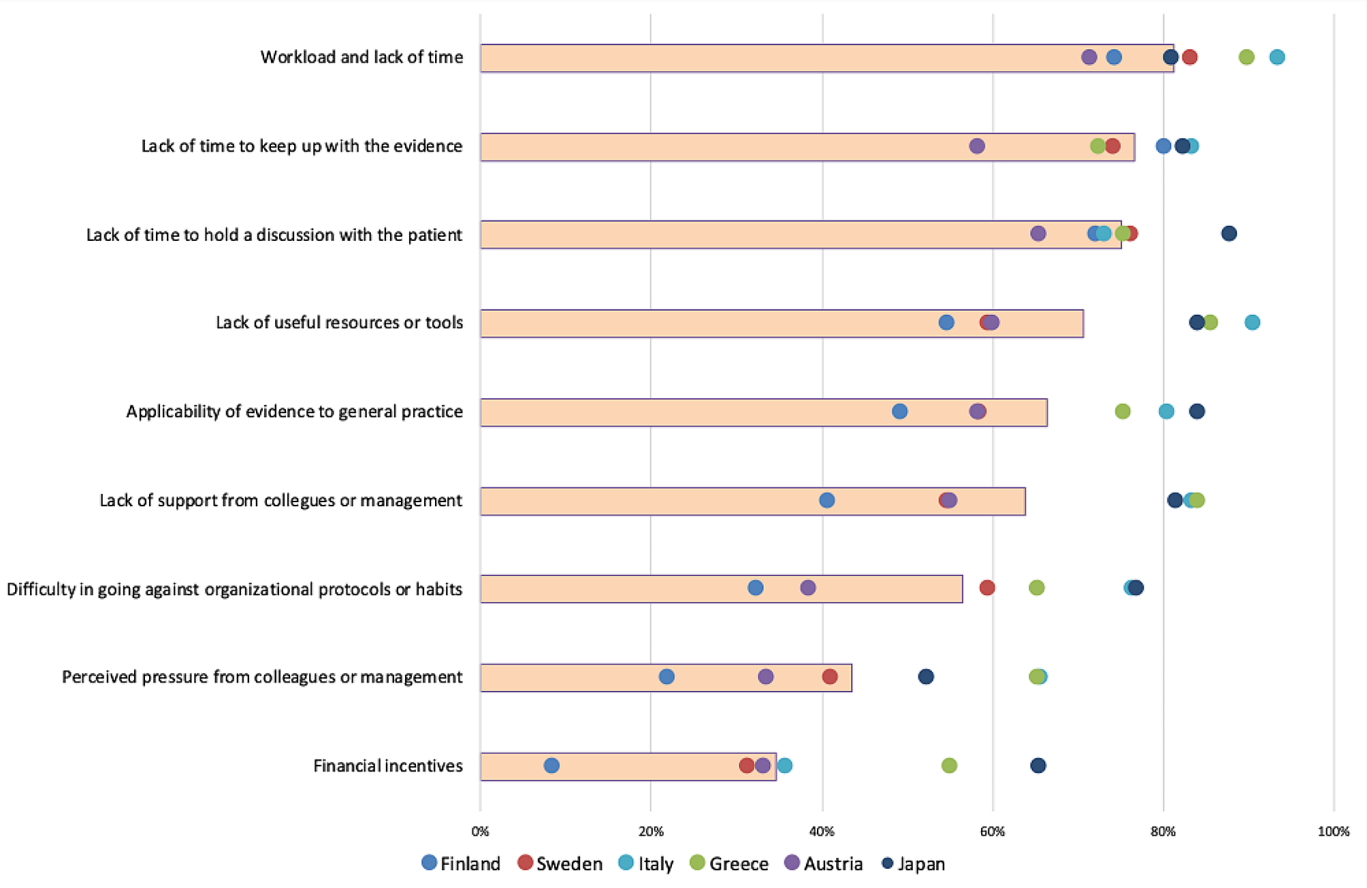
Organizational barriers: proportion (%) of moderate or major importance responses. *The bars represent the proportion of moderate/major responses of all responses and the dots country-specific proportions*

Half of the respondents (52%) rated overdiagnosis as a problem (either to some extent or major) in their own practice, 85% in their country’s health care system, and 83% in other high-income countries (Fig. 1). Respective numbers for overtreatment were 50% (own practice), 81% (own country), and 81% (other high-income countries). In a secondary analysis, we compared answers between respondents’ own practice and their healthcare system; 49% rated overdiagnosis as a bigger problem in their healthcare system, 49% rated it to be a problem of equal magnitude in their practice compared to their country’s healthcare system, and 2% rated overdiagnosis as a bigger problem in their own practice than in their country’s healthcare. Respective numbers for overtreatment were 47%, 51% and 2% (S Figs. 1 and 2). Analysis by work experience is presented in the supplementary file (S Fig. 3).

**Fig. 4:**
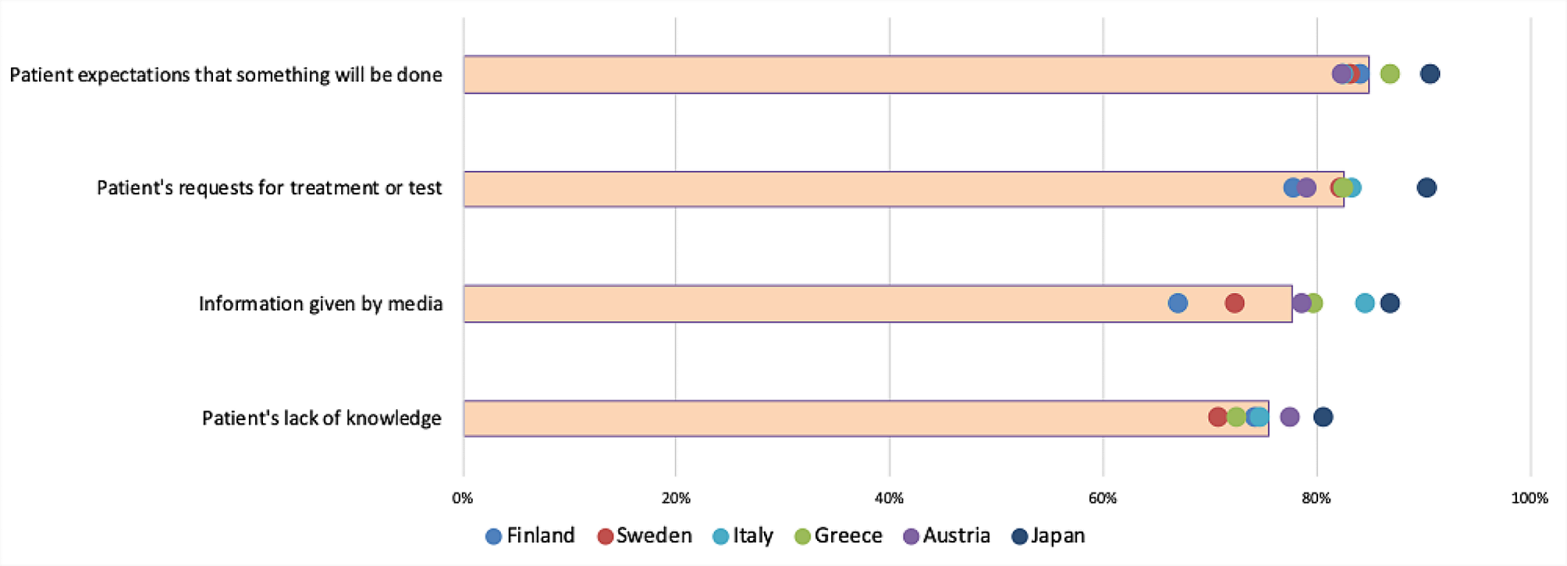
Patient-related barriers: proportion (%) of moderate or major importance responses. *The bars represent the proportion of moderate/major responses of all responses and the dots country-specific proportions*

The three most important individual barriers for de-implementation were (i) fear of medical error (81% of the 1,220 respondents answered either moderate or major importance), (ii) fear of underdiagnosis or undertreatment (79%), and (iii) desire to meet patient expectations (77%) (Fig. 2). The three most important organizational barriers were (i) workload and lack of time (81%), (ii) lack of time to keep up with the evidence (77%), and (iii) lack of time to discuss with the patient (75%) (Fig. 3). The three most important patient-related barriers were (i) patient expectations that something will be done (85%), (ii) patients’ requests for treatments and tests (83%), and (iii) information given by the media (78%) (Fig. 4). Figures by work experience and presenting all response options (no/small/moderate/major importance) are presented in the supplementary file (S Figs. 4, 5, 6 and 7).

In the secondary analysis, the perception of overdiagnosis and overtreatment as a problem in their own practice (question 9 and 12) was positively correlated with Choosing Wisely familiarity (η² = 0.016, *p* < 0.001). Statistical difference was observed between genders (male mean 5.23 [scale 3–8] and female mean 5.04, η² = 0.003, *p* = 0.014), and countries (Austria mean 4.61, Finland 5.03, Greece 5.13, Italy 5.63, Japan 5.17, Sweden 5.20, η² = 0.064, *p* < 0.001), but not correlated with work experience (η² = 0.0004, *p* = 0.055).

Mean perception of all barriers was positively correlated with the perception of overdiagnosis and overtreatment as a problem in their own practice (η² = 0.053, *p* < 0.001) and negatively correlated with longer work experience (η² = 0.003, *p* = 0.044). Statistical difference was observed between countries (Austria 2.68, Finland 2.63, Greece 3.12, Italy 3.00, Japan 3.12, Sweden 2.83 [scale 1–4], η² = 0.147, *p* < 0.001), but not between genders (η² = 0.005, *p* = 0.739), and Choosing Wisely familiarity (η² = 0.00003, *p* = 0.078).

In Finland and Austria, patient-related barriers were rated as more important compared to other barriers than in other countries (S Fig. 8). In organizational barriers, Greek and Italian physicians rated lack of support from colleagues or management and lack of useful resources, and Finnish physicians lack of time to keep up with the evidence as more important compared to other countries. In the individual barriers, Japanese physicians rated lack of knowledge of low-value care as being more important compared to other countries.

## Discussion

In our survey of more than 1500 primary care physicians in six high-income countries, more than 80% of primary care physicians consider overtreatment and overdiagnosis as a problem in their country’s healthcare and approximately half in their own practice. Indeed, almost all respondents (98%) perceived overdiagnosis and overtreatment as either a similar or bigger problem in their healthcare system compared to their own practice. The respondents considered patients’ expectations and requests, fear of medical error, as well as workload and lack of time as the most important barriers to reducing the use of low-value care.

### Comparison to previous studies

Previous studies report a considerably smaller number of important barriers to de-implementation [3, 6, 13, 14, 19, 21]. By identifying barriers from previous studies, we formed a comprehensive list of possible barriers. Therefore, we were able to compare the relative importance of these identified barriers. The large number of important barriers suggests that de-implementation could be an even more complex process than previously thought.

In an American primary care survey, physicians’ most cited reasons for overtreatment were fear of malpractice (85% of respondents), and patient pressures/requests (59%) [3]. In a Dutch survey, 76% of physicians cited “maintaining a good relationship with the patient” as a driver for low-value care use; 56% cited time pressures [14]. German primary care physicians considered patient expectations (76%), lack of a primary care system (61%), and defensive medicine (53%) as the main drivers for medical overuse [23]. These results are generally in line with ours. Especially, patient-related barriers and facilitators have been recognized by all studies. Some barriers, e.g. perceived pressure from others and financial incentives, have been considered important in previous studies but were not in our study [19, 24].

To our knowledge, our survey is the first to compare (quantitatively) differences in barriers to reducing the use of low-value care between different countries. Our survey identified major barriers similar from country to country, with some exceptions. We found differences in the importance of organizational barriers, for example, “financial incentives” and “difficulty in going against organizational protocol or habits”. These different ratings could represent differences in healthcare systems. There was also a substantial difference in perception of “lack of trust in the origin of recommendations”, in which 10% of Finnish respondents rated it as at least a moderate barrier compared to 33–55% in other countries.

Our findings concur with a systematic review that explored general practitioners’ barriers to the use of evidence-based medicine (EBM) [20]. This review found that patients’ preferences and expectations, the applicability of evidence to general practice, time pressure, and lack of knowledge and skills are important barriers.

### Implications

Humans often tend to rate themselves above average [25–27]. In our study, 98% of respondents rated overdiagnosis and overtreatment as being at least as big a problem in their country’s healthcare compared to their own practice. Our findings are similar to the previous literature and likely represent the overconfidence bias phenomenon. About half of the respondents perceived overtreatment and overdiagnosis as minor or no problem in their practice. It may also be a barrier to de-implementation if physician does not see de-implementation of low-value care as important in their own practice, but rather sees low-value care as a problem of others. Our findings highlight the need to continue efforts to implement the concept of low-value care, including overdiagnosis and overtreatment, in regular medical discourse, education, and healthcare. In addition, healthcare authorities should give a high priority to the development of new tools and education that support clinicians in the evaluation of their practices.

Our results could help with developing a system change. In all countries, time constraints, both in relation to keeping up with the evidence and discussing with the patients, were considered an important barrier. This should be acknowledged when designing the healthcare system. For example, electronic health records systems should reduce the burden of recording (and not vice versa). Furthermore, longer patient appointments could facilitate discussions and, at the same time, result in more cost-effective health care.

Among the most important barriers were fear of medical error and fear of underdiagnosis and undertreatment. These may be drivers for defensive medicine, not just to avoid malpractice litigations, but to help with perceived uncertainty. The uncertainty could be reduced with well-organized senior support for younger physicians. Acknowledging why overtreatment and overdiagnosis are often higher risk for the patient than undertreatment and underdiagnosis, could further help in reducing use of low-value care.

Patient-related barriers were perceived as very important in all countries, which might explain why engaging patients in decision-making has proven to be effective in reducing the use of low-value care [28]. Changing from authoritative care decisions to shared decision-making could be supported with education and time for patient encounters.

In addition to the observation of multiple barriers to de-implementation, the process of de-implementation is often complex and multifactorial [29, 30], and the importance of different barriers might vary by context (Figs. 2, 3 and 4). Our results underline the importance of recognizing the local barriers to change and using this knowledge in planning de-implementation strategies. A strategy, copied from another context or setting, might not lead to optimal results. Similarly, understanding only part of the barriers could lead to the development of strategies that fail because an essential part of the change process remains unrecognized. Our comprehensive list of potential barriers could help in finding all important local barriers to change.

There are several differences in how primary care is organized between the countries, including funding, responsibilities and the type of providers (e.g. education, specialization status). This likely explains some of the differences in importance of barriers as well as attitudes towards overdiagnosis and overtreatment. Nevertheless, these changes did not translate into large differences in the most important barriers.

A large number of important barriers and difficulties in de-implementation also highlight the need to implement system changes in which low-value practices are not available (e.g. regulation). If a low-value treatment does not enter the market, physicians are unable to use it, and we will avoid the complexities of de-implementation. For example, having a more restrictive drug approval system or public funding for healthcare services could help in decreasing the number of available low-value practices.

### Strengths and limitations

The recruitment process was adapted to meet the local circumstances and led to some differences in the sample between countries (S Tables 1, 2, 3, 4, 5 and 6). However, according to our analysis, the respondents represented well the real primary care physician population in each country. The biggest difference was in how well younger doctors were reached. Due to these limitations, small differences in the importance of barriers between countries should be interpreted with caution.

Second, the low response rate decreases the generalizability of the results. We chose to use e-mail lists to ensure consistent recruitment procedures due to unavailability of address information in some countries and mailing costs, although this probably led to lower response rate [31]. Selection bias is common in surveys. Those interested in the subject tend to participate. Selection bias cannot be ruled out and may further decrease the generalizability.

Third, only the English version was piloted and translated into local languages by professional translators. This could limit the comparability of the results between the countries. However, the local researchers, who are active in the international Choosing Wisely network and familiar with terms related to low-value care revised the survey if needed. To adjust for the potential response bias, we also compared the importance of different barriers to the mean importance of all barriers (S Fig. 8).

Our multinational survey also has strengths. We carefully prepared the survey with several rounds of discussion among the researchers. In addition, we piloted the questionnaire. We defined the low-value care-related terms in the questionnaire to facilitate the common understanding among the respondents. Over 1500 physicians participated leading to sufficient statistical power, although comparability of included countries could be limited. Due to the large sample, we think that our results represent well the overall importance of attitudes and barriers to de-implementation.

## Conclusions

Primary care physicians largely perceive low-value care as a problem; however, more for others than for themselves. Lack of time, fear of medical error, and patient’s expectations/requests were perceived as the most important barriers to de-implementation and should be acknowledged when planning future healthcare. Differences between countries in perception of organizational and individual barriers highlight the importance of understanding the local barriers in planning de-implementation strategies.

### Electronic supplementary material

Below is the link to the electronic supplementary material.

Supplementary Material 1

Supplementary Material 2

## Data Availability

The datasets generated during and/or analysed during the current study are available from the corresponding author on reasonable request.
