# Supplementary material for: Perspectives on low-value care and barriers to de-implementation among primary care physicians: a multinational survey
Source: BMC Prim Care. 2024 May 9;25:159. doi: 10.1186/s12875-024-02382-9 (PMC11084097; doi:10.1186/s12875-024-02382-9)
Supplement: Supplementary file 1 — Supplementary Material 1 [file 12875_2024_2382_MOESM1_ESM.docx]

# Appendix

**Table of Contents**

1. **S Methods 1.** The survey in English 2
2. **S Tables 1-6.** Sample characteristics 6
3. **S Tables 7-15.** Sample representativeness 15
4. **S Figures 1 and 2.** Attitudes – own practice versus country comparison 20
5. **S Figure 3.** Attitudes – per age group 21
6. **S Figures 4-6.** Barriers by age group 22
7. **S Figure 7.** Barriers – Complete scale of answers 24
8. **S Figure 8.** Barriers – Mean answers comparison 25

**Survey on low-value care in primary care**

We are a multidisciplinary team of clinical researchers conducting a multinational survey on low-value care and de-implementation. The survey is led by researchers from the University of Helsinki and the Finnish Medical Society Duodecim in Finland. Thank you for helping with this initiative!

**Background information**

| 1. | Age: ☐ <30 ☐ 30–39 ☐ 40–49 ☐ 50–59 ☐ ≥60 | | | | | | | | | | | | | | |
| --- | --- | --- | --- | --- | --- | --- | --- | --- | --- | --- | --- | --- | --- | --- | --- |
| 2. | Gender: | | ☐ Male | | | | | ☐ Female | | | | ☐ Other | | | |
| 3. | Have you been in primary care clinical practice during the last 24 months? | | | | | | | | | | | | | | |
| ☐ Yes | | | | | ☐ No (*if you answered “No,” please stop answering the survey and return the survey to us*) | | | | | | | | | |  |
| 4. | During the last 24 months, what proportion of your work time was dedicated to clinical work? | | | | | | | | | | | | | | |
| ☐ 0–20% | | ☐ 21–40% | | | | ☐ 41–60% | | | | ☐ 61–80% | | | ☐ 81–100% | | |
| 5. | How long have you been working in clinical practice? | | | | | | | | | | | | | | |
| ☐ <5 years | | | | ☐ 5–10 years | | | ☐ 11–20 years | | | | ☐ 21–30 years | | | ☐ ≥31 years | |
| 6. | What is your specialization status? | | | | | | | | | | | | | | |
| ☐ Resident/in training in general/family medicine | | | | | | | | | ☐ Consultant/specialized in general/family medicine | | | | | | |
| ☐ Resident/in training in occupational health | | | | | | | | | ☐ Consultant/specialized in occupational health | | | | | | |
| ☐ No specialization | | | | | | | | | ☐ Other, please specify _______________ | | | | | | |

**Familiarity with the Choosing Wisely recommendations**

Please answer the next two questions about your familiarity with the Choosing Wisely recommendations. Choose the closest option.

| 7. | Are you familiar with the Choosing Wisely recommendations? | | | |
| --- | --- | --- | --- | --- |
| I have never heard of them ☐ | | I have heard of them ☐ | I have read a few ☐ | I have read many ☐ |
| 8. | Do you follow Choosing Wisely recommendations which are relevant in your own clinical practice? | | | |
| Never ☐ | | Rarely ☐ | Often ☐ | Always ☐ |

**General questions about overdiagnosis and overtreatment**

Below are three statements about overdiagnosis and three statements about overtreatment. Please complete the sentences by choosing one option from 1 to 4. Overdiagnosis refers to 1) the diagnosis of a medical condition that would never have caused any symptoms or problems, or to 2) medicalizing ordinary life experiences through expanded definitions of diseases. Overdiagnosis can be caused by overdetection or overdefinition of disease. Overtreatment refers to treatment for which there is no or little benefit to the patient, considering both the potential harm from, and benefit of, the treatment.

| 9. In my practice, overdiagnosis is _______? | | | |
| --- | --- | --- | --- |
| 1. not a problem at all ☐ | 2. a minor problem ☐ | 3. a problem to some extent ☐ | 4. a major problem ☐ |
| 10. In the Finnish health-care system, overdiagnosis is _______? | | | |
| 1. not a problem at all ☐ | 2. a minor problem ☐ | 3. a problem to some extent ☐ | 4. a major problem ☐ |
| 11. In other high-income countries, overdiagnosis is_______? | | | |
| 1. not a problem at all ☐ | 2. a minor problem ☐ | 3. a problem to some extent ☐ | 4. a major problem ☐ |
|  |  |  |  |
| 12. In my practice, overtreatment is _______? | | | |
| 1. not a problem at all ☐ | 2. a minor problem ☐ | 3. a problem to some extent ☐ | 4. a major problem ☐ |
| 13. In the Finnish health-care system, overtreatment is _______? | | | |
| 1. not a problem at all ☐ | 2. a minor problem ☐ | 3. a problem to some extent ☐ | 4. a major problem ☐ |
| 14. In other high-income countries, overtreatment is_______? | | | |
| 1. not a problem at all ☐ | 2. a minor problem ☐ | 3. a problem to some extent ☐ | 4. a major problem ☐ |

**If you answered “not a problem at all” to both questions 9 and 12, please stop answering here and return the survey to us.**

**Barriers to reducing the use of low-value care**

| **15. Barriers related to health-care professional** | **No importance** | **Small importance** | **Moderate importance** | **Major importance** |
| --- | --- | --- | --- | --- |
| Lack of knowledge of low-value care | ☐ | ☐ | ☐ | ☐ |
| Fear of medical error | ☐ | ☐ | ☐ | ☐ |
| Fear of underdiagnosis/undertreatment | ☐ | ☐ | ☐ | ☐ |
| Feeling that avoiding low-value care is not important | ☐ | ☐ | ☐ | ☐ |
| Routines and habits | ☐ | ☐ | ☐ | ☐ |
| Lack of communicational skills (to convince patient about treatment/test harmfulness) | ☐ | ☐ | ☐ | ☐ |
| Uncertainty or disagreement on what not to do | ☐ | ☐ | ☐ | ☐ |
| Lack of trust in the origin of the recommendation(s) | ☐ | ☐ | ☐ | ☐ |
| Difficulty finding (trustworthy) information on low-value care | ☐ | ☐ | ☐ | ☐ |
| Desire to meet patient expectations | ☐ | ☐ | ☐ | ☐ |
| **16. Organizational barriers** | | | | |
| Workload and lack of time | ☐ | ☐ | ☐ | ☐ |
| Lack of support from colleagues or management | ☐ | ☐ | ☐ | ☐ |
| Lack of useful resources or tools (e.g., for shared decision-making) | ☐ | ☐ | ☐ | ☐ |
| Applicability of evidence to general practice | ☐ | ☐ | ☐ | ☐ |
| Lack of time to keep up with the evidence | ☐ | ☐ | ☐ | ☐ |
| Perceived pressure from colleagues or management | ☐ | ☐ | ☐ | ☐ |
| Lack of time to hold a discussion with the patient | ☐ | ☐ | ☐ | ☐ |
| Difficulty in going against organizational protocols or habits | ☐ | ☐ | ☐ | ☐ |
| Financial incentives | ☐ | ☐ | ☐ | ☐ |
| **17. Patient-related barriers** | | | | |
| Patient’s expectations that something will be done | ☐ | ☐ | ☐ | ☐ |
| Patient’s lack of knowledge | ☐ | ☐ | ☐ | ☐ |
| Patient’s requests for treatment or test | ☐ | ☐ | ☐ | ☐ |
| Information given by the media | ☐ | ☐ | ☐ | ☐ |

Below we list potential barriers to reducing the use of low-value care in clinical practice. We ask you to evaluate how important each individual barrier is in **your own clinical practice**. Low-value care refers to medical practices that are unlikely to benefit the patient given the potential harm or cost of the treatment, available alternatives, or preferences of a patient.

**How to reduce low-value care practices**

Your responses are important when considering future de-implementation in Finland. We greatly appreciate your efforts!

18. In your opinion, which low-value care practices is it most important to reduce/abandon in the Finnish health-care system?

19. What would encourage or help you to reduce the use of low-value care?

*Please describe any type of influences that would encourage you to reduce the use of low-value care. What could your colleagues, organizations and society (e.g. your workplace, medical societies, the government) do to help you reduce the use of low-value care?*

S Table 1. Japan sample characteristics

| Target sample size, that is, number of invited physicians | Sample size ：10,500 （assumed response rate of 5%） |
| --- | --- |
| Describe your target sample (to whom you will send the survey), including:   1. primary care/secondary care 2. specialized/non-specialized 3. workplaces 4. demographics | Accurate data is not officially announced.  i) mainly primary care  ii) both specialized and non-specialized (there are no established requirements to ensure the quality of primary care physicians)  iii) mainly clinics and community hospitals  iv) no data available |
| From where and how did you get the sample? | We plan to sample all physician members of the Japan Primary Care Association. |
| Representativeness: are there reasons in your sample why it may not be representative of your country’s primary care physicians? | In Japan, many accreditation agencies operate independently and there are no established requirements or systems to ensure the quality of primary care physicians. In many practices, primary care is currently provided by semi-generalists/semi-specialists (see OECD 2014 reports). Therefore, it was difficult to conduct a random sampling of all primary care physicians. |
| How did you distribute the survey? | Using a mailing list of Japan Primary Care Association physician members |
| Who was the sender of the invitation email? | Yuki Kaji and Japan Primary Care Association staffs. |
| What survey platform did you use? | SurveyMonkey |

S Table 2. Finland characteristics

| Target sample size, that is, number of invited physicians | 2000 |
| --- | --- |
| Describe your target sample (to whom you will send the survey), including:   1. primary care/secondary care 2. specialized/non-specialized 3. workplaces 4. demographics | - Mainly primary care. For non-specialized physicians, we could not separate between primary care and secondary care but we had the ratio for how many were in primary/secondary care. Therefore, we increased our sample size to include 2000 primary care physicians. - Both specialized and non-specialized - Primary care health centers ~80%, ~20% in occupational health centers - Random sample from the whole country |
| From where and how did you get the sample? | Sample was from Finnish Medical Association database. |
| Representativeness: are there reasons in your sample why it may not be representative of your country’s primary care physicians? | The sample represents well the primary care physician population in finland |
| How did you distribute the survey? | Finnish medical association sent the emails. |
| Who was the sender of the invitation email? | The finnish medical association |
| What survey platform did you use? | SurveyMonkey |

S Table 3. Sweden characteristics

| Target sample size, that is, number of invited physicians | 2313 |
| --- | --- |
| Describe your target sample (to whom you will send the survey), including:   1. primary care/secondary care 2. specialized/non-specialized 3. workplaces 4. demographics | - The sample included only physicians working in primary care, although there could be some delays in the updates of the workplace - Includes specialists, residents and physicians that are not specializing - The private company invites physicians to the database – and includes about 2362/6570 primary care physicians |
| From where and how did you get the sample? | Private company database (IQVIA) |
| Representativeness: are there reasons in your sample why it may not be representative of your country’s primary care physicians? | The sample represents all parts of the primary care system, although there could be a bias, as all primary care physicians are not included in the database. |
| How did you distribute the survey? | The company sent all emails. |
| Who was the sender of the invitation email? | The company (IQVIA) |
| What survey platform did you use? | SurveyMonkey |

S Table 4. Greece characteristics

| Target sample size, that is, number of invited physicians | 745 |
| --- | --- |
| Describe your target sample (to whom you will send the survey), including:   1. primary care/secondary care 2. specialized/non-specialized 3. workplaces 4. demographics | Primary care/secondary care  specialized/non-specialized |
| From where and how did you get the sample? | List of physicians from Internal Medicine Society of Greece |
| Representativeness: are there reasons in your sample why it may not be representative of your country’s primary care physicians? | Νο |
| How did you distribute the survey? | By email |
| Who was the sender of the invitation email? | Secretary of Internal Medicine Society of Greece |
| What survey platform did you use? | Survey Monkey |

S Table 5. Austria characteristics

| Target sample size, that is, number of invited physicians | 3813 |
| --- | --- |
| Describe your target sample (to whom you will send the survey), including:   1. primary care/secondary care 2. specialized/non-specialized 3. workplaces 4. demographics | - Primary care physicians mostly, sample included also physicians with several specializations, who could also be working in secondary care - Primary care health centers and individual practices |
| From where and how did you get the sample? | Austrian Medical Association as GPs |
| Representativeness: are there reasons in your sample why it may not be representative of your country’s primary care physicians? | Νο. Still GPs working in hospital setting were not included. This includes also most young doctors as physicians have to complete specialization before they can have an individual practice. |
| How did you distribute the survey? | By email |
| Who was the sender of the invitation email? | - |
| What survey platform did you use? | Limesurvey |

S Table 6. Italy characteristics

| Target sample size, that is, number of invited physicians | 2500 |
| --- | --- |
| Describe your target sample (to whom you will send the survey), including:   1. primary care/secondary care 2. specialized/non-specialized 3. workplaces 4. demographics | - Primary care physicians working in Tuscany region, including both specialized and non-specialized GPs |
| From where and how did you get the sample? | Local investigators had access to emails of 116 territorial coordinators. Emails were sent to them and then distributed to GPs by the territorial coordinators. |
| Representativeness: are there reasons in your sample why it may not be representative of your country’s primary care physicians? | Responses may not represent the whole Italy. Also it is possible that territorial coordinators did not distribute the emails to their local GPs and therefore all GPs from Tuscany were not contacted. |
| How did you distribute the survey? | By email. |
| Who was the sender of the invitation email? | Local investigators. |
| What survey platform did you use? | “Homemade” |

**S Table 7. Finland physician age and gender distributions**

| Age | Real distribution | Survey |
| --- | --- | --- |
| 20-29 | 16,23 % | 14,06 % |
| 30-39 | 41,77 % | 31,07 % |
| 40-49 | 15,51 % | 20,86 % |
| 50-59 | 17,22 % | 19,27 % |
| 60+ | 9,27 % | 14,74 % |
|  |  |  |
| Gender |  |  |
| Men | 37,27 % | 37,41 % |
| Women | 62,73 % | 62,59 % |

**S Table 8. Japan physician age and gender distributions**

| Age | Real distribution | Survey |
| --- | --- | --- |
| 20-29 | 4,63 % | 2,78 % |
| 30-39 | 21,31 % | 32,64 % |
| 40-49 | 30,09 % | 31,25 % |
| 50-59 | 24,00 % | 17,71 % |
| 60+ | 19,98 % | 15,63 % |
|  |  |  |
| Sex |  |  |
| Men | 80,09 % | 71,43 % |
| Women | 19,79 % | 28,57 % |

**S Table 9. Italy physician age and gender distributions**

| Age | Real distribution | Survey |
| --- | --- | --- |
| 20-29 | 0,9 % | 1,57 % |
| 30-39 | 12,8 % | 21,57 % |
| 40-49 | 9,3 % | 11,76 % |
| 50-59 | 14,0 % | 16,08 % |
| 60+ | 63,0 % | 49,02 % |
|  |  |  |
| Sex |  |  |
| Men | 59,10 % | 61,18 % |
| Women | 40,90 % | 38,82 % |

**In other countries, real distributions were not available. Distributions were discussed with the investigators and results are in following tables.**

# S Table 10. Finland sample representation

| **Topic** | **Explanation** |
| --- | --- |
| Age | The survey results represent well the real age distribution of primary care physicians in Finland. |
| Clinical work | Represents the real situation in Finland as most are working full time in clinical fork. |
| Experience | More junior physicians than in other countries. Represents the real situation in the Finnish healthcare where most young physicians go first to primary care after graduation. |
| Specialization | Represents well the real situation in Finnish healthcare. |

# S Table 11. Austria sample representation

| **Topic** | **Explanation** |
| --- | --- |
| Age | People can’t go to work in (outpatient) primary care before specialization. Young doctors usually go to work in the hospital at first and single-handled practices just afterward (when more confident?) |
| Clinical work | Corona pandemic might have an effect as the work was done more on paper. |
| Experience | This represents the actual situation. |
| Specialization | Most are GPs but there is also small proportion of other specialties that work in primary care. The survey was sent only to doctors that were specialized. (GPs working in hospitals were excluded). |

# S Table 12. Greece sample representation

| **Topic** | **Explanation** |
| --- | --- |
| Age | After graduation, a lot of physicians move to other countries. -> less young physicians in primary care in Greece. |
| Clinical work | Physicians do a lot of paper work (prescribing for chronic diseases etc.). Some see patients just a little. System limits the time for seeing the patients. Does not represent part time jobs. |
| Experience | After education finished. A lot of physicians move to other countries. -> less young physicians in primary care in Greece. |
| Specialization | No specializing physicians. The distribution had them, but 0 answers. Might be due to large workload related to COVID. Most specialist in primary care are internists. |

# S Table 13. Italy Sample representation

| **Topic** | **Explanation** |
| --- | --- |
| Age | Most GPs in Italy are over 60 also in real population. |
| Clinical work | Most are in full time jobs |
| Experience | Same as Age. |
| Specialization | Italy has specific training course for GPs, which is not specialization (mandatory for younger GPs, Born after -65). |

# S Table 14. Sweden sample representation

| **Topic** | **Explanation** |
| --- | --- |
| Age | More young people have started to enter primary care, but it is still difficult to get young people to the primary care. Often they enter the primary care after hospital service. Might be that there is bias towards older people. The sample (database) did not include so many young doctors. |
| Clinical work | Sample seems representative of the situation. |
| Experience | There is some bias towards older people. Young doctors are not added to the database at the moment of graduation. |
| Specialization | Same issue than in previous section. There is very small group of physicians that do not specialize, so that partly explains why no answers. |

# S Table 15. Japan sample representation

| **Topic** | **Explanation** |
| --- | --- |
| Age | The survey results generally represent the real age distribution of primary care physicians who are members of the Japanese Primary Care Association. |
| Clinical work | It is not clear whether this survey represents the real situation of primary care physicians in Japan, since the percentage of members of the Japanese Primary Care Association who work full-time in clinical have not been surveyed. |
| Experience | Less senior physicians than in other countries. This represents the real situation in the Japan healthcare where primary care physicians are a relatively new profession in Japan. |
| Specialization | It is not clear whether this survey represents the real situation of primary care physicians in Japan, since the percentage of specialties among the members of the Japanese Primary Care Association has not been surveyed. |

**S Figure 1.** Attitudes towards overtreatment. *Results by comparing individual responder’s answers on country and own practice levels.*


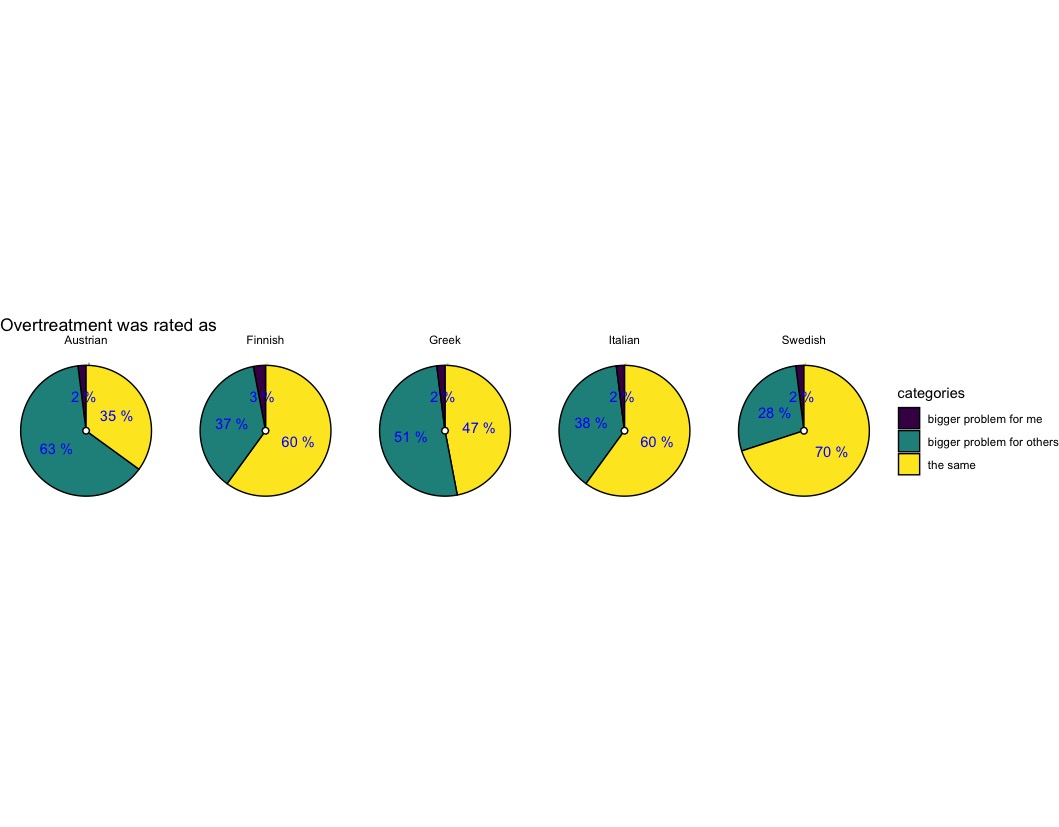


**S Figure 2.** Attitudes towards overdiagnosis. *Results by comparing individual responder’s answers on country and own practice levels.*


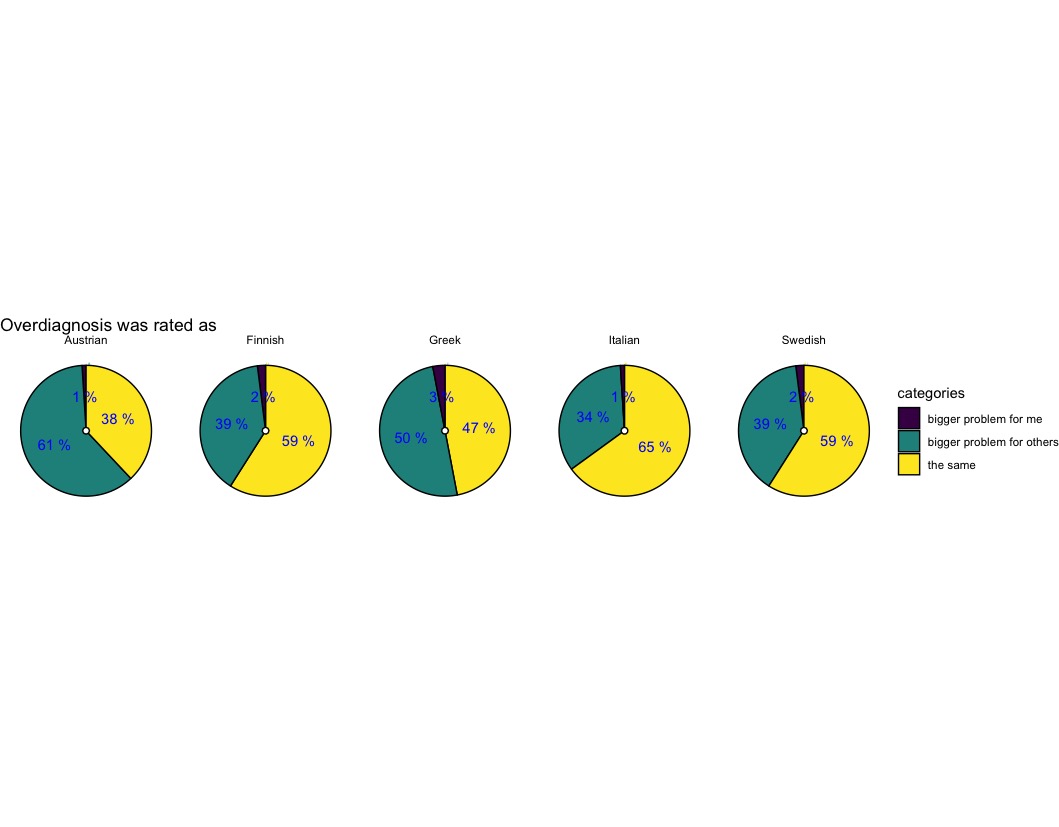


**S Figure 3.** Attitudes towards overdiagnosis and overtreatment

| **Overdiagnosis** | **Overtreatment** |
| --- | --- |
| In my practice | |
|  |  |
| In my country’s healthcare system | |
|  |  |
| In other high-income countries | |
|  |  |

**S Figure 4.** Individual barriers by age group

**S Figure 5.** Organizational barriers by age group

**S Figure 6.** Patient-related barriers by age group

**Figure 7.** Barriers – all answer options

**Figure 8.** Mean answer in comparison to mean. *The bars represent country-specific mean answers – country specific mean of all barriers.*


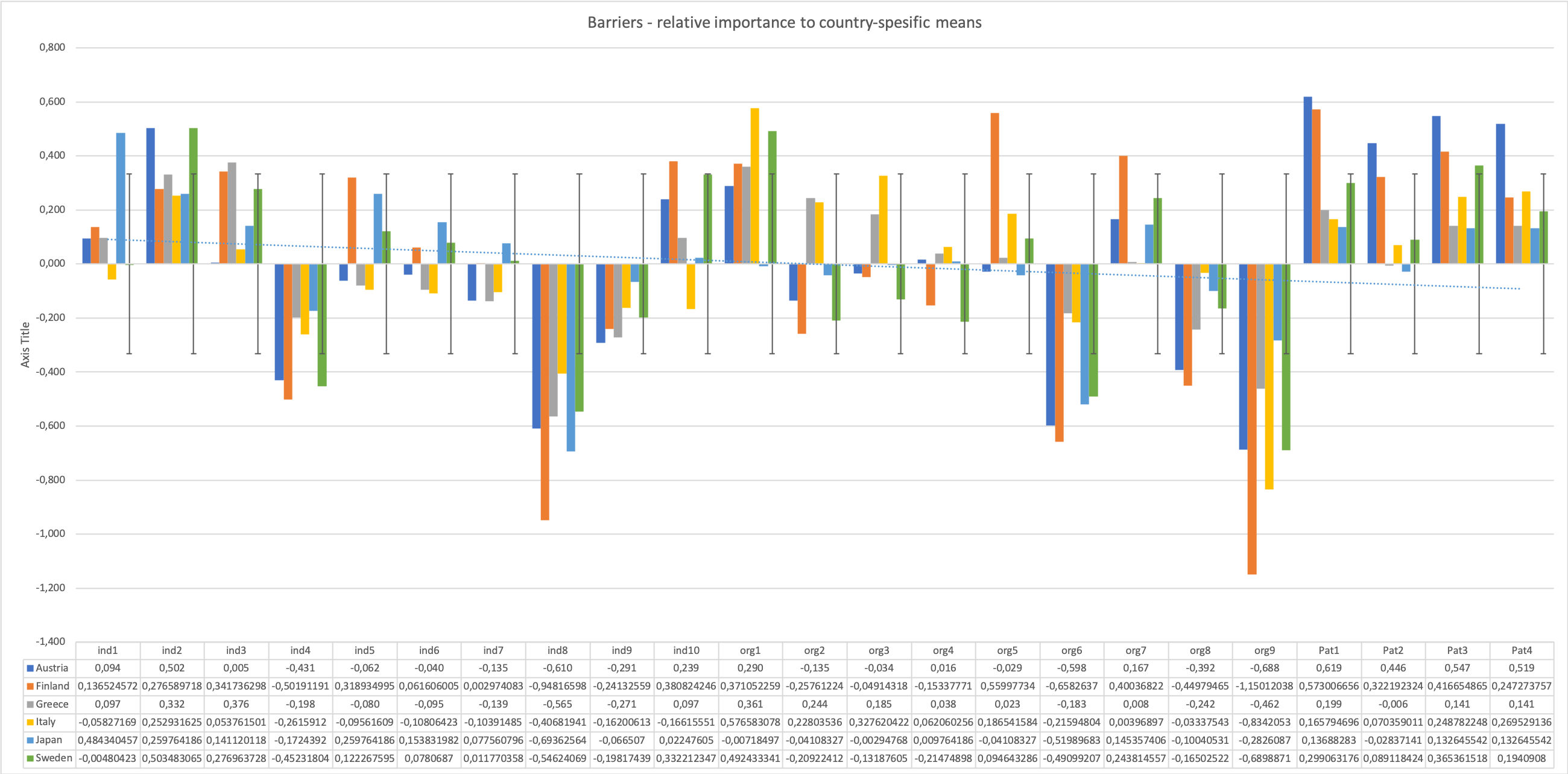


| Ind1 = lack of knowledge of low-value care  Ind2 = fear of medical error  Ind3 = fear of underdiagnosis/undertreatment  Ind4 = feeling that avoiding low-value care is not important  Ind5 = routines and habits  Ind6 = lack of communication skills  Ind7 = uncertainty and disagreements on what not to do  Ind8 = lack of trust in the origin of the recommendation  Ind9 = difficulty finding trustworthy information on low-value care  Ind10 = desire to meet patient expectations | Org1 = workload and lack of time  Org2 = lack of support from colleagues or management  Org3 = lack of useful resources (e.g. for shared decision making)  Org4 = applicability of ecidence to general practice  Org5 = lack of time to keep up with the evidence  Org6 = perceived pressure from colleagues or management  Org7 = lack of time to hold a discussion with the patient  Org8 = difficulty in going against organizational protocols and habits  Org9 = financial incentives | Pat1 = patient’s expectations that something will be done  Pat2 = patient’s lack of knowledge  Pat3 = patient’s requests for treatment or test  Pat4 = Information given by the media |
| --- | --- | --- |
