# Supplementary material for: Perspectives on low-value care and barriers to de-implementation among primary care physicians: a multinational survey
Source: BMC Prim Care. 2024 May 9;25:159. doi: 10.1186/s12875-024-02382-9 (PMC11084097; doi:10.1186/s12875-024-02382-9)
Supplement: Supplementary file 2 — Supplementary Material 2 [file 12875_2024_2382_MOESM2_ESM.docx]

**Kysely vähähyötyisistä hoidoista perusterveydenhuollossa**

Olemme monitieteinen tutkijaryhmä ja teemme monikansallista tutkimusta vähähyötyisestä hoidosta ja sen vähentämisestä terveydenhuollossa. Suomessa tutkimusta johtavat Helsingin yliopiston ja Suomalaisen Lääkäriseura Duodecimin tutkijat. Kiitos osallistumisesta!

**Taustatietoa**

| 1. | Ikä: ☐ <30 ☐ 30–39 ☐ 40–49 ☐ 50–59 ☐ ≥60 | | | | | | | | | | | | | |
| --- | --- | --- | --- | --- | --- | --- | --- | --- | --- | --- | --- | --- | --- | --- |
| 2. | Sukupuoli: | | ☐ Mies | | | | ☐ Nainen | | | | ☐ Muu | | | |
| 3. | Oletko työskennellyt perusterveydenhuollossa viimeisten 24 kuukauden aikana? | | | | | | | | | | | | | |
| ☐ Kyllä | | | | | ☐ Ei | | | | | | | | |  |
| 4. | Kuinka suuri osuus työajastasi oli viimeisten 24 kuukauden aikana kliinistä työtä? | | | | | | | | | | | | | |
| ☐ 0–20% | | ☐ 21–40% | | | | ☐ 41–60% | | | ☐ 61–80% | | | ☐ 81–100% | | |
| 5. | Kuinka kauan olet työskennellyt kliinisessä työssä? | | | | | | | | | | | | | |
| ☐ <5 vuotta | | | | ☐ 5–10 vuotta | | | ☐ 11–20 vuotta | | | ☐ 21–30 vuotta | | | ☐ ≥31 vuotta | |
| 6. | Mikä on erikoisalasi? | | | | | | | | | | | | | |
| ☐ Erikoistuva lääkäri - yleislääketiede | | | | | | | | ☐ Erikoislääkäri - yleislääketiede | | | | | | |
| ☐ Erikoistuva lääkäri - työterveys | | | | | | | | ☐ Erikoislääkäri - työterveys | | | | | | |
| ☐ Ei erikoistumista | | | | | | | | ☐ Muu, tarkenna _______________ | | | | | | |

**Vältä Viisaasti -suositukset**

Vastaa kahteen seuraavaan kysymykseen siitä, miten hyvin tunnet Vältä Viisaasti -suositukset. Valitse sopivinvaihtoehto.

| 7. | Tunnetko Vältä Viisaasti -suositukset? | | | |
| --- | --- | --- | --- | --- |
| En koskaan ole kuullut niistä ☐ | | Olen kuullut niistä ☐ | Olen lukenut muutaman ☐ | Olen lukenut monta ☐ |
| 8. | Noudatatko Vältä viisaasti -suosituksia, jotka ovat merkityksellisiä omassa kliinisessä työssäsi? | | | |
| En koskaan ☐ | | Harvoin ☐ | Usein ☐ | Aina ☐ |

**Yleisiä kysymyksiä ylidiagnostiikasta ja ylihoidosta**

Alla on kolme väittämää ylidiagnostiikasta ja kolme ylihoidosta. Täydennä lauseet valitsemalla yksi neljästä vaihtoehdosta. Ylidiagnostiikalla tarkoitetaan 1) sellaisen sairauden diagnosointia, joka ei olisi koskaan aiheuttanut mitään oireita tai haittaa, tai 2) tavallisten elämänkokemusten medikalisaatiota(lääketieteellistämistä) sairauksien laajennettujen määritelmien avulla. Ylidiagnosointi voi johtua sairauden liiallisesta havaitsemisesta tai liiallisesta määrittelystä. Ylihoidolla tarkoitetaan hoitoa, josta on vähän tai ei lainkaan hyötyä potilaalle, kun otetaan huomioon sekä hoidosta mahdollisesti aiheutuvat haitat että siitä saatava hyöty.

| 9. Omassa työskentelyssäni ylidiagnostiikka _______? | | | |
| --- | --- | --- | --- |
| 1. ei ole lainkaan ongelma ☐ | 2. on pieni ongelma ☐ | 3. on jonkin verran ongelma ☐ | 4. on suuri ongelma ☐ |
| 10. Suomalaisessa terveydenhuoltojärjestelmässä ylidiagnostiikka _______? | | | |
| 1. ei ole lainkaan ongelma ☐ | 2. on pieni ongelma ☐ | 3. on jonkin verran ongelma ☐ | 4. on suuri ongelma ☐ |
| 11. Muissa korkean tulotason maissa ylidiagnostiikka _______? | | | |
| 1. ei ole lainkaan ongelma ☐ | 2. on pieni ongelma ☐ | 3. on jonkin verran ongelma ☐ | 4. on suuri ongelma ☐ |
|  |  |  |  |
| 12. Omassa työskentelyssäni ylihoito _______? | | | |
| 1. ei ole lainkaan ongelma ☐ | 2. on pieni ongelma ☐ | 3. on jonkin verran ongelma ☐ | 4. on suuri ongelma ☐ |
| 13. Suomalaisessa terveydenhuoltojärjestelmässä ylihoito _______? | | | |
| 1. ei ole lainkaan ongelma ☐ | 2. on pieni ongelma ☐ | 3. on jonkin verran ongelma ☐ | 4. on suuri ongelma ☐ |
| 14. Muissa korkean tulotason maissa ylihoito _______? | | | |
| 1. ei ole lainkaan ongelma ☐ | 2. on pieni ongelma ☐ | 3. on jonkin verran ongelma ☐ | 4. on suuri ongelma ☐ |

**Jos vastasit kysymyksiin 9 ja 12 "ei ole lainkaan ongelma", lopeta vastaaminen tähän ja palauta kysely meille.**

**Vähähyötyisen hoidon vähentämisen esteet**

Seuraavassa luetellaan mahdollisia esteitä vähähyötyisen hoidon käytön vähentämiselle terveydenhuollossa. Arvioi kuinka merkittävä kukin yksittäinen este on **omassa kliinisessä työssäsi**. Vähähyötyisellä hoidolla tarkoitetaan hoitokäytäntöjä, jotka eivät todennäköisesti hyödytä potilasta, kun otetaan huomioon hoidon mahdollinen haitta, kustannukset, käytettävissä olevat vaihtoehdot ja potilaan mieltymykset.

| **15. Terveydenhuollon ammattilaisiin liittyvät esteet** | **Ei merkitystä** | **Pieni merkitys** | **Kohtalainen merkitys** | **Suuri merkitys** |
| --- | --- | --- | --- | --- |
| Vähähyötyistä hoitoa koskevan tiedon puute | ☐ | ☐ | ☐ | ☐ |
| Hoitovirheiden pelko | ☐ | ☐ | ☐ | ☐ |
| Pelko alidiagnostiikasta/alihoidosta | ☐ | ☐ | ☐ | ☐ |
| Tunne siitä, että vähähyötyisen hoidon välttäminen ei ole tärkeää | ☐ | ☐ | ☐ | ☐ |
| Rutiinit ja tavat | ☐ | ☐ | ☐ | ☐ |
| Viestintätaitojen puute (potilaan vakuuttaminen hoidon/testin haitallisuudesta) | ☐ | ☐ | ☐ | ☐ |
| Epävarmuus tai erimielisyys siitä, mitä ei pidä tehdä | ☐ | ☐ | ☐ | ☐ |
| Luottamuksen puute suosituksen (suositusten) alkuperään | ☐ | ☐ | ☐ | ☐ |
| Vaikeus löytää (luotettavaa) tietoa vähähyötyisestä hoidosta | ☐ | ☐ | ☐ | ☐ |
| Halu vastata potilaan odotuksiin | ☐ | ☐ | ☐ | ☐ |
| **16. Organisaatioon liittyvät esteet** | | | | |
| Työmäärä ja ajan puute | ☐ | ☐ | ☐ | ☐ |
| Kollegoiden tai johdon tuen puute | ☐ | ☐ | ☐ | ☐ |
| Hyödyllisten resurssien tai välineiden puute (esim. jaettuun päätöksentekoon) | ☐ | ☐ | ☐ | ☐ |
| Näytön sovellettavuus perusterveydenhuoltoon | ☐ | ☐ | ☐ | ☐ |
| Ajan puute perehtyä tutkimusnäyttöön | ☐ | ☐ | ☐ | ☐ |
| Kollegoiden tai johdon aiheuttama paine | ☐ | ☐ | ☐ | ☐ |
| Ajan puute käydä keskustelu potilaan kanssa. | ☐ | ☐ | ☐ | ☐ |
| Vaikeus toimia vastoin organisaation protokollaa tai käytäntöjä | ☐ | ☐ | ☐ | ☐ |
| Taloudelliset kannustimet | ☐ | ☐ | ☐ | ☐ |
| **17. Potilaaseen liittyvät esteet** | | | | |
| Potilaan odotukset siitä, että jotain tehdään | ☐ | ☐ | ☐ | ☐ |
| Potilaan tiedon puute | ☐ | ☐ | ☐ | ☐ |
| Potilaan pyynnöt hoidosta tai testeistä | ☐ | ☐ | ☐ | ☐ |
| Tiedotusvälineiden antamat tiedot | ☐ | ☐ | ☐ | ☐ |

**Vähäarvoisten hoitokäytäntöjen vähentäminen**

Vastauksesi ovat tärkeitä, kun suunnitellaan vähähyötyisen hoidon vähentämistä Suomessa. Arvostamme suuresti ajankäyttöäsi!

18. Mitä hoitokäytäntöjä olisi tärkeintä vähentää ja mistä mahdollisesti kokonaan luopua suomalaisessa terveydenhuoltojärjestelmässä?

19. Mikä kannustaisi tai auttaisi sinua vähentämään vähähyötyisten hoitojen käyttöä?

*Kuvaile vaikutteita, jotka kannustaisivat sinua vähentämään vähähyötyisen hoidon käyttöä. Mitä kollegasi, organisaatiot ja yhteiskunta (esim. työpaikkanne, lääkäriseurat, hallitus) voisivat tehdä auttaakseen sinua vähentämään vähähyötyisen hoidon käyttöä?*

**プライマリ・ケアにおける低価値医療(low-value care)に関する意識調査：国際共同研究**

この度、プライマリ・ケア連合学会医療の質・患者安全委員会では、ヘルシンキ大学およびフィンランド医学会を中心とした国際的な研究グループ（スウェーデン、ドイツ、オーストリア、イタリア、ギリシャ、日本、イスラエル）とともに実施する国際、学会員の医師を対象としたプライマリ・ケアにおける低価値医療（Low-value care）についての意識調査を実施することとなりました。低価値医療とは、利益と害、利用可能な代替案、コスト、患者の嗜好などを考慮したうえでも、患者にとっての利益がほとんど与えられない医療行為を指します。この問題は近年さらに注目されており、みなさまのご意見をお聞かせいただければ幸いです。

なお、本調査ではプライマリ・ケアにおける国際共同研究を進めるとともに、学会事業の一環として今後のプライマリ・ケア連合学会医療の質・患者安全委員会におけるChoosing Wiselyの推奨事項作成とその普及・実装のための基礎資料とすることを目的としています。

※ここでのプライマリ・ケアとは、一般内科外来および小児科外来といった初療にあたる全ての診療の場を含み、診療所だけではなく病院の診療科としての外来も含まれます。

【倫理審査について】

この研究は日本プライマリ・ケア連合学会での倫理審査委員会で審査・承認され実施されます

【質問紙調査について】

本調査への協力は自由意志であり、ご協力いただけない場合でも今後の活動には一切影響することはありません。Webの質問紙の提出をもって、本研究への参加の同意を得たものとします。なお、本調査は無記名で実施するため、提出後に同意の撤回を希望されても、回答者の特定が困難であるため回答を除外することはできません。

【個人情報の取り扱いについて】

回収した調査結果は、研究機関（国際医療福祉大学）およびヘルシンキ大学・フィンランド医学会が研究目的のみに利用します。また、その他の機関への提供することはありません。研究の成果は学会や専門雑誌などの発表に使用される場合がありますが、個人を特定されるような情報は公表されることはありません。

以下、主任研究者Aleksi J Raudasojaからのメッセージになります。

私たちは、低価値医療（low value care）とそれを減らす介入（de-implementation）に関して多国間での調査を行っている臨床研究者の学際的チームです。この調査は、ヘルシンキ大学とフィンランド医師会が中心となり、日本プライマリ・ケア連合学会の協力のもと実施しています。この度は、本調査にご協力いただき誠に感謝申し上げます。

| 問１．あなたの年齢: | ☐30歳未満　　☐30-39歳　　☐40-49歳　　☐50-59歳　☐60歳以上 |
| --- | --- |
| 問２．あなたの性別: | ☐ 男性　☐ 女性 ☐ その他 |
| 問３．あなたは過去24ヶ月間、プライマリ・ケアの現場で勤務されていましたか？ | |
| ☐ はい  ☐いいえ（「いいえ」と答えた方は、ここでアンケートへの回答を中止し、送信してください）。 | |
| 問４．過去24ヶ月間、あなたの仕事時間のうち、臨床業務に従事していた割合はどのくらいですか？ | |
| ☐ 0-20%　　☐ 21-40%　　☐ 41-60%　　☐ 61-80%　　☐ 81-100% | |
| 問５．積極的に（プライマリ・ケアの現場で）臨床業務を行っていた期間はどのくらいですか？ | |
| ☐ 5年未満　　☐ 5-10年　　☐ 11-20 年　　☐ 21-30 年　　☐ 31年以上 | |
| 問６．あなたの専門分野は何ですか？ （複数回答可） | |
| ☐ 家庭医療の研修医・専攻医  ☐ 家庭医療の専門医・一般医師  ☐ 病院総合診療（病院総合内科）の研修医・専攻医  ☐ 病院総合診療（病院総合内科）の専門医・一般医師  ☐ 小児医療の研修医・専攻医  ☐ 小児医療の専門医・一般医師  ☐ 産業医学の研修医・専攻医  ☐ 産業医学の専門医・一般医師  ☐ 専門領域なし  ☐ その他 – 具体的には ______________________ | |

Choosing Wiselyキャンペーンの推奨について、以下の２つの質問に回答してください。最も近いと考える選択肢を選んでください。

| 問７．あなたはChoosing Wiselyキャンペーンの推奨についてご存じですか？ | | | |
| --- | --- | --- | --- |
| 全く聞いたことがない☐ | 聞いたことはある ☐ | 少しだけ読んだことがある ☐ | 何度も読んだことがある ☐ |
| 問８．あなたは自分の診療に関連するChoosing Wiselyキャンペーンの推奨に沿った診療を行っていますか？ | | | |
| まったく行っていない☐ | たまに行っている☐ | しばしば行っている☐ | 常に行っている ☐ |

以下に、過剰診断に関する３つの文章と過剰治療に関する3つの文章をお示しします。1～4の中から選択肢を１つ選んで、文章を完成させてください。

ここでの「過剰診断（Overdiagnosis）」とは、１）本来は何の症状や問題も生じない病状に対して診断をつけること、２）病気の定義を拡大解釈することによって、ごく普通の生活体験を医療の対象とする（＝医療化する）こと、を意味しています。過剰診断は、検査などで病気を過剰に検出したり、過剰に定義したりすることで生じます。

また、ここでの「過剰治療（Overtreatment）」とは、潜在的な利益と害のバランスを考慮したうえで、患者にとっての利益がわずか、あるいは全くない治療、を意味しています。

| 問９．自分の臨床現場では、過剰診断は _______ | | | |
| --- | --- | --- | --- |
| 1. 全く  問題になっていない ☐ | 2. あまり  問題になっていない ☐ | 3. ある程度問題と  なっている ☐ | 4. 大きな問題と  なっている ☐ |
| 問10．日本の医療システムでは、過剰診断は_______ | | | |
| 1. 全く  問題になっていない ☐ | 2. あまり  問題になっていない ☐ | 3. ある程度問題と  なっている ☐ | 4. 大きな問題と  なっている ☐ |
| 問11．他の高所得国では、過剰診断は_______ | | | |
| 1. 全く  問題になっていない ☐ | 2. あまり  問題になっていない ☐ | 3. ある程度問題と  なっている ☐ | 4. 大きな問題と  なっている ☐ |
|  |  |  |  |
| 問12．自分の臨床現場では、過剰治療は_______ | | | |
| 1. 全く  問題になっていない ☐ | 2. あまり  問題になっていない ☐ | 3. ある程度問題と  なっている ☐ | 4. 大きな問題と  なっている ☐ |
| 問13．日本の医療システムでは、過剰治療は_______ | | | |
| 1. 全く  問題になっていない ☐ | 2. あまり  問題になっていない ☐ | 3. ある程度問題と  なっている ☐ | 4. 大きな問題と  なっている ☐ |
| 問14．他の高所得国では、過剰治療は_______ | | | |
| 1. 全く  問題になっていない ☐ | 2. あまり  問題になっていない ☐ | 3. ある程度問題と  なっている ☐ | 4. 大きな問題と  なっている ☐ |

問９と問12の両方に「全く問題にならない」と答えた方は、ここまでで回答をやめてアンケートを返送してください。

| **問15．医療従事者に関する阻害要因** | **全く重要ではない** | **あまり重要ではない** | **ある程度**  **重要である** | **非常に**  **重要である** |
| --- | --- | --- | --- | --- |
| 低価値医療についての知識不足 | ☐ | ☐ | ☐ | ☐ |
| 医療過誤に対する不安 | ☐ | ☐ | ☐ | ☐ |
| 過少診断/過少治療に対する不安 | ☐ | ☐ | ☐ | ☐ |
| 低価値医療を避けることは重要ではないという気持ち | ☐ | ☐ | ☐ | ☐ |
| ルーティーンと習慣 | ☐ | ☐ | ☐ | ☐ |
| （治療・検査の有害性を患者に納得させるための）コミュニケーション能力の欠如 | ☐ | ☐ | ☐ | ☐ |
| 実施するべきでない医療についての不確実性や見解の相違 | ☐ | ☐ | ☐ | ☐ |
| （Choosing Wiselyの）推奨の発信元に対する信頼感の欠如 | ☐ | ☐ | ☐ | ☐ |
| 低価値医療に関する（信頼性の高い）情報を見つけることの難しさ | ☐ | ☐ | ☐ | ☐ |
| 患者さんの期待・希望に応えてあげたいという気持ち | ☐ | ☐ | ☐ | ☐ |
| **問16**．**組織に関する阻害要因** | | | | |
| 仕事量が過多/時間の欠如 | ☐ | ☐ | ☐ | ☐ |
| 同僚や経営陣からのサポート不足 | ☐ | ☐ | ☐ | ☐ |
| 有用なリソースやツールの不足（例：共有意思決定のためのツール） | ☐ | ☐ | ☐ | ☐ |
| エビデンスを日常診療で実施する際の適用可能性の問題 | ☐ | ☐ | ☐ | ☐ |
| 最新のエビデンスを把握するための時間が足りない | ☐ | ☐ | ☐ | ☐ |
| 同僚や経営陣からのプレッシャーを感じる | ☐ | ☐ | ☐ | ☐ |
| 患者と十分に話し合うための時間不足 | ☐ | ☐ | ☐ | ☐ |
| 組織の取り決めや慣習に逆らうことの難しさ | ☐ | ☐ | ☐ | ☐ |
| 経済的なインセンティブ | ☐ | ☐ | ☐ | ☐ |
| **問17**．**患者に関する阻害要因** | | | | |
| 患者からの「何かをしてほしい」という期待 | ☐ | ☐ | ☐ | ☐ |
| 患者の知識不足 | ☐ | ☐ | ☐ | ☐ |
| 患者の治療や検査への要求 | ☐ | ☐ | ☐ | ☐ |
| メディアから受ける情報 | ☐ | ☐ | ☐ | ☐ |

以下は、臨床現場における低価値医療を減らす際に妨げとなる要因をリストアップしたものです。それぞれの要因について、**ご自身の臨床現場で**低価値医療を減らす際にどの程度重要であるかを評価してください。

ここでの「低価値医療（Low-value care）」とは、有害性、コスト、利用可能な代替手段、患者の選好などを考慮すると、患者の利益になりそうにない医療行為、を意味しています。

次に、日本で低価値医療を減らしていくことを検討する上で皆様からのご意見をお聞かせください。ご協力いただき感謝します。

問19．低価値医療の削減を推奨/手助けしていくためには何があればよいでしょうか？（自由回答）

※低価値医療を減らすのに影響するあらゆる要因について記載してください。低価値医療を減らすために、同僚、組織、社会（職場や学会、政府など）にはどのようなことをしてほしいですか？

問18．日本の医療システムにおいて、どのような低価値な医療行為を削減/放棄することが重要だと考えますか？あなたのご意見で構いません（自由回答）

**Enkät om lågvärdig vård i primärvården**

Vi är ett multidisciplinärt team av kliniska forskare som genomför en multinationell undersökning om lågvärdig vård och de-implementering. Undersökningen leds av forskare från Helsingfors universitet och Finska Läkaresällskapet Duodecim i Finland, i samarbete med yngre läkare från Stockholm, Sverige. Tack för att du hjälper till med detta initiativ!

**Bakgrundsinformation**

| 1. | Ålder: ☐ <30 ☐ 30–39 ☐ 40–49 ☐ 50–59 ☐ ≥60 | | | | | | | | | | | | | | |
| --- | --- | --- | --- | --- | --- | --- | --- | --- | --- | --- | --- | --- | --- | --- | --- |
| 2. | Kön: | | ☐ Man | | | | | ☐ Kvinna | | | | ☐ Övrig | | | |
| 3. | Har du varit kliniskt aktiv inom primärvården under de senaste 24 månaderna? | | | | | | | | | | | | | | |
| ☐ Ja | | | | | ☐ Nej (*om du svarade “Nej,” vänligen sluta svara på enkäten och returnera enkäten till oss)* | | | | | | | | | |  |
| 4. | Hur stor del av din arbetstid har ägnats åt kliniskt arbete under de senaste 24 månaderna? | | | | | | | | | | | | | | |
| ☐ 0–20% | | ☐ 21–40% | | | | ☐ 41–60% | | | | ☐ 61–80% | | | ☐ 81–100% | | |
| 5. | Hur länge har du arbetat på klinisk praktik? | | | | | | | | | | | | | | |
| ☐ <5 år | | | | ☐ 5–10 år | | | ☐ 11–20 år | | | | ☐ 21–30 år | | | ☐ ≥31 år | |
| 6. | Vilken är din specialiseringsstatus? | | | | | | | | | | | | | | |
| ☐ ST-läkare/innan ST inom allmänmedicin | | | | | | | | | ☐ Specialist inom allmänmedicin | | | | | | |
| ☐ ST-läkare/innan ST inom inom arbetsmedicin/företagshälsåvård | | | | | | | | | ☐ Specuialist inom inom inom arbetsmedicin/företagshälsåvård | | | | | | |
| ☐ Ingen inriktning | | | | | | | | | ☐ Annan, vänligen specificera _______________ | | | | | | |

**Erfarenhet av ‘Kloka Listan’ rekommendationerna**

Vänligen svara på de två kommande frågorna angående din erfarenhet med ”Kloka listan” rekommendationerna. Välj närmaste alternativ.

| 7. | Har du erfarenhet av Kloka listan rekommendationerna? | | | |
| --- | --- | --- | --- | --- |
| Jag har aldrig hört talas om dem ☐ | | Jag har hört talats om dem ☐ | Jag har läst några ☐ | Jag har läst många ☐ |
| 8. | Följer du Kloka listan rekommendationerna som är relevanta i din egen klinisk praktik? | | | |
| Aldrig ☐ | | Sällan ☐ | Ofta ☐ | Alltid ☐ |

**Allmänna frågor om överdiagnostik och överbehandling**

Nedan finns tre påstående om överdiagnostik och tre påståenden om överbehandling. Vänligen fullför meningarna genom att välja ett alternativ från 1 till 4. Överdiagnostik avser, 1). Diagnos av ett medicinskt tillstånd som aldrig skulle ha orsakat några symptom eller problem, eller 2). Medikalisering av vanliga livserfarenheter genom utökande definitioner av sjukdomar. Överdiagnostik kan orsakas av överupptäckt eller överdefiniering av sjukdom. Överbehandling avser behandling där det inte finns någon eller liten nytta för patienten, med tanke på både den potentiella skadan och nyttan av behandlingen

| 9. I min praktik är överdiagnostik _______? | | | |
| --- | --- | --- | --- |
| 1. Inget problem☐ | 2. Ett mindre problem ☐ | 3. Ett problem till viss del ☐ | 4. Ett stort problem ☐ |
| 10. I den svenska sjukvården är överdiagnostik _______? | | | |
| 1. Inget problem ☐ | 2. Ett mindre problem ☐ | 3. Ett problem till viss del ☐ | 4. Ett stort problem ☐ |
| 11. I andra höginkomstländer är överdiagnostik_______? | | | |
| 1. Inget problem ☐ | 2. Ett litet problem ☐ | 3. Ett problem till viss del ☐ | 4. Ett stort problem ☐ |
|  |  |  |  |
| 12. I min praktik är överbehandling _______? | | | |
| 1. Inget problem ☐ | 2. Ett mindre problem ☐ | 3. Ett problem till viss del ☐ | 4. Ett stort problem ☐ |
| 13. I den svenska sjukvården är överbehandling _______? | | | |
| 1. Inget problem ☐ | 2. Ett mindre problem ☐ | 3. Ett problem till viss del ☐ | 4. Ett stort problem ☐ |
| 14. I andra höginkomstländer är överbehandling_______? | | | |
| 1. Inget problem alls ☐ | 2. Ett mindre problem ☐ | 3. Ett problem till viss del ☐ | 4. Ett stort problem ☐ |

**Om du svarade “Inget problem alls” på både frågorna 9 och 12, vänligen sluta svara här och lämna tillbaka enkäten till oss.**

| **15. Hinder relaterade till vårdpersonal** | **Ingen betydelse** | **Liten betydelse** | **Måttlig betydelse** | **Stor betydelse** |
| --- | --- | --- | --- | --- |
| Bristande kunskap om lågvärdig vård | ☐ | ☐ | ☐ | ☐ |
| Rädsla av medicinska misstag | ☐ | ☐ | ☐ | ☐ |
| Rädsla för underdiagnostik/underbehandling | ☐ | ☐ | ☐ | ☐ |
| Känna att det inte är viktigt att undvika lågvärdig vård | ☐ | ☐ | ☐ | ☐ |
| Rutiner och vanor | ☐ | ☐ | ☐ | ☐ |
| Brist på kommunikationsförmåga (för att övertyga patienten om behandling/testskadlighet) | ☐ | ☐ | ☐ | ☐ |
| Osäkerhet eller oenighet om vad man inte ska göra | ☐ | ☐ | ☐ | ☐ |
| Bristande förtroende för ursprunget till rekommendationen/rekommendationerna | ☐ | ☐ | ☐ | ☐ |
| Svårighet i att hitta (pålitlig) information om lågvärdig vård | ☐ | ☐ | ☐ | ☐ |
| Önskan att möta patienternas förväntningar | ☐ | ☐ | ☐ | ☐ |
| **16. Organisatoriska hinder** | | | | |
| Arbetsbelastning och tidsbrist | ☐ | ☐ | ☐ | ☐ |
| Brist med stöd från kollegor och eller ledning | ☐ | ☐ | ☐ | ☐ |
| Brist på användbara resurser eller verktyg (till exempel för delat beslutsfattande) | ☐ | ☐ | ☐ | ☐ |
| Tillämplighet av bevis i allmän praxis | ☐ | ☐ | ☐ | ☐ |
| Tidsbrist för att hålla jämna steg med bevisen | ☐ | ☐ | ☐ | ☐ |
| Upplevd press från kollegor eller ledning | ☐ | ☐ | ☐ | ☐ |
| Tidsbrist för att föra samtal med patienten | ☐ | ☐ | ☐ | ☐ |
| Svårigheter att gå emot organisatoriska protokoll eller vanor | ☐ | ☐ | ☐ | ☐ |
| Ekonomiska incitament | ☐ | ☐ | ☐ | ☐ |
| **17. Patientrelaterade hinder** | | | | |
| Patientens förväntning att något kommer att göras | ☐ | ☐ | ☐ | ☐ |
| Patientens brist av kunskap | ☐ | ☐ | ☐ | ☐ |
| Patientens önskemål om behandling eller test | ☐ | ☐ | ☐ | ☐ |
| Information som ges av media | ☐ | ☐ | ☐ | ☐ |

**Hinder för att minska användningen av lågvärdig vård**

Nedan listar vi potentiella hinder för att minska användningen av lågvärdig vård i klinisk praxis. Vi ber dig att utvärdera hur viktig varje enskilt hinder är **i din egen kliniska praktik**. Vård med lågt värde avser medicinsk praxis som sannolikt inte kommer att gynna patienten med tanke på den potentiella skadan eller kostnaden för behandlingen, tillgängliga alternativ eller preferenser hos en patient.

**Hur man minskar lågvärda vårdpraxis**

Dina svar är viktiga när man överväger de-implementering i Sverige i framtiden. Vi uppskattar dina ansträngningar mycket!

18. Vilken lågvärdig vård är enligt din mening viktigast att minska eller ta bort i den svenska sjukvården?

19. Vad skulle uppmuntra eller hjälpa dig att minska användningen av lågvärdig vård?

*Vänligen beskriv alla typer av influenser som skulle uppmuntra dig att minska användningen av lågvärdig vård. Vad kan dina kollegor, organisationer, och samhället (till exempel din arbetsplats, medicinska föreningar, regeringen) göra för att hjälpa dog att minska användningen av lågvärdig vård?*

**Έρευνα για τη φροντίδα “low-value” (χαμηλής ανταποδοτικής αξίας) στην Φροντίδα Υγείας των ασθενών.**

Είμαστε μια διεπιστημονική ομάδα κλινικών ερευνητών που διεξάγουμε μια πολυκεντρική μελέτη με θέμα την φροντίδα χαμηλής ανταποδοτικής αξίας (“low-value care”). Η έρευνα αυτή πραγματοποιείται από ερευνητές του «University of Helsinki» και από την «Finnish Medical Society Duodecim» στη Φιλανδία, σε συνεργασία με την Εταιρεία Παθολογίας Ελλάδας, στην Ελλάδα. Σας ευχαριστούμε που συνεισφέρετε σε αυτή τη πρωτοβουλία!

**Βασικές πληροφορίες**

| 1. | Ηλικία: ☐ <30 ☐ 30–39 ☐ 40–49 ☐ 50–59 ☐ ≥60 | | | | | | | | | | | | | | |
| --- | --- | --- | --- | --- | --- | --- | --- | --- | --- | --- | --- | --- | --- | --- | --- |
| 2. | Φύλο: | | ☐ Αρσενικό | | | | | ☐ Θηλυκό | | | | ☐ Άλλο | | | |
| 3. | Συμμετείχατε στην κλινική φροντίδα υγείας ασθενών κατά τη διάρκεια των τελευταίων 24 μηνών; | | | | | | | | | | | | | | |
| ☐ Ναι | | | | | ☐ Όχι (*αν απαντήσατε «Όχι», παρακαλούμε μη συμπληρώσετε την υπόλοιπη έρευνα*) | | | | | | | | | |  |
| 4. | Κατά τη διάρκεια των τελευταίων 24 μηνών, τι ποσοστό της δουλειάς σας αφιερώθηκε σε κλινική πρακτική; | | | | | | | | | | | | | | |
| ☐ 0–20% | | ☐ 21–40% | | | | ☐ 41–60% | | | | ☐ 61–80% | | | ☐ 81–100% | | |
| 5. | Πόσο καιρό εργάζεστε στη κλινικά; | | | | | | | | | | | | | | |
| ☐<5 χρόνια | | | | ☐5–10 χρόνια | | | ☐11–20 χρόνια | | | | ☐21–30 χρόνια | | | ☐≥31 χρόνια | |
| 6. | Ποια είναι η βαθμίδα σας; | | | | | | | | | | | | | | |
| ☐ Ειδικευόμενος ιατρός εσωτερικής παθολογίας/ γενικής ιατρικής | | | | | | | | | ☐ Ειδικός ιατρός εσωτερικής παθολογίας/γενικής ιατρικής | | | | | | |
| ☐ Ειδικευόμενος γενικής ιατρικής | | | | | | | | | ☐ Ειδικός ιατρός Εργασίας | | | | | | |
| ☐ Καμία ειδικότητα | | | | | | | | | ☐ Άλλο, παρακαλώ προσδιορίστε _______________ | | | | | | |

**Εξοικείωση με τις συστάσεις «Choosing Wisely» (Επιλέγοντας σοφά)**

Παρακαλούμε απαντήστε τις επόμενες δύο ερωτήσεις σχετικά με την εξοικείωση σας με τις συστάσεις «Choosing Wisely». Διαλέξτε την κοντινότερη επιλογή.

| 7. | Είστε εξοικειωμένοι με τις συστάσεις «Choosing Wisely»; | | | |
| --- | --- | --- | --- | --- |
| Δεν τις έχω ακούσει ☐ | | Έχω ακούσει για αυτές ☐ | Έχω διαβάσει κάποιες ☐ | Έχω διαβάσει αρκετές ☐ |
| 8. | Ακολουθείτε τις συστάσεις « Choosing Wisely» που είναι σχετικές με τη δική σας κλινική πρακτική; | | | |
| Ποτέ ☐ | | Σπάνια ☐ | Συχνά ☐ | Πάντα ☐ |

**Γενικές ερωτήσεις για την «υπερ-διάγνωση» (overdiagnosis) και την «υπερ-θεραπεία» (overtreatment)**

Παρακάτω παρατίθενται τρεις προτάσεις για την «υπερδιάγνωση» και τρεις προτάσεις για την «υπερθεραπεία». Παρακαλούμε συμπληρώστε τις προτάσεις διαλέγοντας μια από τις επιλογές 1 έως 4.

Η «υπερδιάγνωση» αναφέρεται 1) στην διάγνωση μιας ιατρικής κατάστασης που δεν θα προκαλούσε ποτέ συμπτώματα ή προβλήματα ή 2) στην «ιατρικοποίηση» συνηθισμένων καταστάσεων της ζωής μέσω διεύρυνσης των νοσολογικών ορισμών. Η «υπερδιάγνωση» μπορεί να οφείλεται στην «υπερβολική ανίχνευση» ή στην «υπερβολική διεύρυνση ορισμού» της νόσου.

Η «υπερθεραπεία» αναφέρεται στην θεραπεία που έχει ελάχιστο ή καθόλου όφελος για τον ασθενή, λαμβάνοντας υπόψιν τόσο την πιθανή βλάβη, όσο και το όφελος από αυτή.

| 9. Στην πρακτική μου, η «υπερδιάγνωση» _______; | | | |
| --- | --- | --- | --- |
| 1. δεν είναι καθόλου πρόβλημα☐ | 2. είναι μικρό πρόβλημα ☐ | 3. είναι πρόβλημα έως ένα βαθμό ☐ | 4. είναι σημαντικό πρόβλημα ☐ |
| 10. Στο Ελληνικό σύστημα υγείας, η υπερδιάγνωση _______; | | | |
| 1. δεν είναι καθόλου πρόβλημα ☐ | 2. είναι μικρό πρόβλημα ☐ | 3. είναι πρόβλημα έως ένα βαθμό ☐ | 4. είναι σημαντικό πρόβλημα ☐ |
| 11. Σε άλλες χώρες υψηλού εισοδήματος, η υπερδιάγνωση _______; | | | |
| 1. δεν είναι καθόλου πρόβλημα ☐ | 2. είναι μικρό πρόβλημα ☐ | 3. είναι πρόβλημα έως ένα βαθμό ☐ | 4. είναι σημαντικό πρόβλημα ☐ |
|  |  |  |  |
| 12. . Στην πρακτική μου, η «υπερθεραπεία» _______; | | | |
| 1. δεν είναι καθόλου πρόβλημα ☐ | 2. είναι μικρό πρόβλημα ☐ | 3. είναι πρόβλημα έως ένα βαθμό ☐ | 4. είναι σημαντικό πρόβλημα ☐ |
| 13. Στο Ελληνικό σύστημα υγείας, η υπερθεραπεία _______; | | | |
| 1. δεν είναι καθόλου πρόβλημα ☐ | 2. είναι μικρό πρόβλημα ☐ | 3. είναι πρόβλημα έως ένα βαθμό ☐ | 4. είναι σημαντικό πρόβλημα ☐ |
| 14. Σε άλλες χώρες υψηλού εισοδήματος, η υπερθεραπεία _______; | | | |
| 1. δεν είναι καθόλου πρόβλημα ☐ | 2. είναι μικρό πρόβλημα ☐ | 3. είναι πρόβλημα έως ένα βαθμό ☐ | 4. είναι σημαντικό πρόβλημα ☐ |

**Αν απαντήσατε «δεν είναι καθόλου πρόβλημα» τόσο στην ερώτηση 9 όσο και στην ερώτηση 12, παρακαλούμε σταματήστε σε αυτό το σημείο να συμπληρώνετε την έρευνα.**

**Εμπόδια στην ελάττωση χρήσης της φροντίδας “low-value”**

| **15. Εμπόδια σχετιζόμενα με τους επαγγελματίες υγείας** | **Καμίας σημασίας** | **Μικρής σημασίας** | **Μέτριας σημασίας** | **Μεγάλης σημασίας** |
| --- | --- | --- | --- | --- |
| Έλλειψη επίγνωσης της φροντίδας “low-value” | ☐ | ☐ | ☐ | ☐ |
| Φόβος για ιατρικό λάθος | ☐ | ☐ | ☐ | ☐ |
| Φόβος για ελλιπή διάγνωση/θεραπεία | ☐ | ☐ | ☐ | ☐ |
| Αίσθηση ότι η αποφυγή της θεραπείας “low-value” δεν είναι σημαντική | ☐ | ☐ | ☐ | ☐ |
| Ρουτίνα και συνήθειες | ☐ | ☐ | ☐ | ☐ |
| Έλλειψη επικοινωνιακών δεξιοτήτων (μη ικανότητα πειθούς σχετικά με τις πιθανές βλάβες της θεραπείας/εξέτασης) | ☐ | ☐ | ☐ | ☐ |
| Αβεβαιότητα ή διαφωνία για το τι δεν πρέπει να γίνει | ☐ | ☐ | ☐ | ☐ |
| Έλλειψη εμπιστοσύνης στη πηγή προέλευσης των συστάσεων (κατευθυντήριων οδηγιών) | ☐ | ☐ | ☐ | ☐ |
| Δυσκολία στην εύρεση (αξιόπιστων) πληροφοριών για την φροντίδα “low-value” | ☐ | ☐ | ☐ | ☐ |
| Επιθυμία για ικανοποίηση των προσδοκιών του ασθενή | ☐ | ☐ | ☐ | ☐ |
| **16. Οργανωτικά εμπόδια** | | | | |
| Φόρτος εργασίας και έλλειψη χρόνου | ☐ | ☐ | ☐ | ☐ |
| Έλλειψη υποστήριξης από τους συναδέλφους ή τη διοίκηση | ☐ | ☐ | ☐ | ☐ |
| Έλλειψη χρήσιμων πόρων ή εργαλείων (π.χ. για κοινή λήψη αποφάσεων) | ☐ | ☐ | ☐ | ☐ |
| Εφαρμοσιμότητα των οδηγιών στη κλινική πρακτική | ☐ | ☐ | ☐ | ☐ |
| Έλλειψη χρόνου για εφαρμογή των οδηγιών | ☐ | ☐ | ☐ | ☐ |
| Αισθητή πίεση από τους συναδέλφους ή από τη διοίκηση | ☐ | ☐ | ☐ | ☐ |
| Έλλειψη χρόνου για συζήτηση με τον ασθενή | ☐ | ☐ | ☐ | ☐ |
| Δυσκολία στην μη εφαρμογή των πρωτοκόλλων ή των πρακτικών | ☐ | ☐ | ☐ | ☐ |
| Οικονομικά κίνητρα | ☐ | ☐ | ☐ | ☐ |
| **17. Εμπόδια που σχετίζονται με τον ασθενή** | | | | |
| Προσδοκίες του ασθενούς ότι κάτι επιπλέον θα γίνει | ☐ | ☐ | ☐ | ☐ |
| Έλλειψη γνώσεων από τον ασθενή | ☐ | ☐ | ☐ | ☐ |
| Αιτήματα του ασθενή για θεραπεία/εξέταση | ☐ | ☐ | ☐ | ☐ |
| Πληροφορίες παρεχόμενες από τα Μέσα Μαζικής Ενημέρωσης και Κοινωνικής Δικτύωσης | ☐ | ☐ | ☐ | ☐ |

Παρακάτω παραθέτουμε μια λίστα με πιθανά εμπόδια στη μείωση της χρήσης της φροντίδας “low-value” στην κλινική πράξη. Καλείστε να αξιολογήσετε πόσο σημαντικό είναι κάθε αναφερόμενο εμπόδιο στη δική σας κλινική πράξη. Η φροντίδα “low-value” αναφέρεται στις ιατρικές πρακτικές που είναι λιγότερο πιθανό να ωφελήσουν τον ασθενή, δεδομένης της πιθανής βλάβης ή του κόστους της θεραπείας, των διαθέσιμων εναλλακτικών λύσεων και των προτιμήσεων του ασθενή.

**Πως να ελαττωθούν οι πρακτικές φροντίδας “low value”**

Οι απαντήσεις σας είναι σημαντικές, δεδομένου ότι είναι πιθανό να εξετασθεί το ενδεχόμενο της μελλοντικής κατάργησης της εφαρμογής πρακτικών “low-value” στην Ελλάδα. Εκτιμούμε πολύ την συνεισφορά σας!

19. Τι θα σας ενθάρρυνε ή θα σας βοηθούσε να μειώσετε τη χρήση φροντίδας “low value”;

*Παρακαλούμε περιγράψτε οποιουδήποτε είδους επιρροές θα σας ενθάρρυναν να ελαττώσετε τη χρήση φροντίδας* “low value”*. Τι θα μπορούσαν να κάνουν οι συνάδελφοι σας, οι οργανισμοί και η κοινωνία (π.χ. ο χώρος εργασίας σας, οι ιατρικές εταιρίες, η κυβέρνηση) με σκοπό να μειωθεί η χρήση φροντίδας* “low value”*;*

18. Κατά την άποψη σας, ποιες πρακτικές φροντίδας “low value” είναι πιο σημαντικό να ελαττωθούν/εγκαταλειφθούν στο Ελληνικό σύστημα υγειονομικής περίθαλψης;
